# TNFAIP2 confers cisplatin resistance in head and neck squamous cell carcinoma via KEAP1/NRF2 signaling

**DOI:** 10.1186/s13046-023-02775-1

**Published:** 2023-08-01

**Authors:** Teng Xu, Yuemei Yang, Zhihong Chen, Jinsong Wang, Xiaolei Wang, Yang Zheng, Chao Wang, Yachen Wang, Zaiou Zhu, Xu Ding, Junbo Zhou, Gang Li, Hongchuang Zhang, Wei Zhang, Yunong Wu, Xiaomeng Song

**Affiliations:** 1 Department of Oral and Maxillofacial Surgery, Affiliated Hospital of Stomatology, Nanjing Medical University, Nanjing, China; 2 Jiangsu Key Laboratory of Oral Diseases, Nanjing Medical University, Nanjing, China; 3 Jiangsu Province Engineering Research Center of Stomatological Translational Medicine, Nanjing Medical University, Nanjing, China; 4 Department of Pathology, Nanjing First Hospital, Nanjing Medical University, Nanjing, China; 5 Department of Oral Maxillofacial & Head and Neck Oncology, Shanghai Ninth People’s Hospital, Shanghai Jiao Tong University School of Medicine, National Clinical Research Center of Stomatology, Shanghai, China; 6 Department of Stomatology, Nanjing Integrated Traditional Chinese and Western Medicine Hospital, Nanjing, China; 7 Department of Stomatology, Affiliated Hospital of Xuzhou Medical University, Xuzhou, China; 8 Department of Stomatology, Xuzhou No. 1 Peoples Hospital, Xuzhou, China

**Keywords:** Head and neck squamous cell carcinoma, TNFAIP2, KEAP1/NRF2, Oxidative stress, Cisplatin resistant

## Abstract

**Background:**

Drug resistance limits the treatment effect of cisplatin-based chemotherapy in head and neck squamous cell carcinoma (HNSCC), and the underlying mechanism is not fully understood. The aim of this study was to explore the cause of cisplatin resistance in HNSCC.

**Methods:**

We performed survival and gene set variation analyses based on HNSCC cohorts and identified the critical role of tumor necrosis factor alpha-induced protein 2 (TNFAIP2) in cisplatin-based chemotherapy resistance. Half-maximal inhibitory concentration (IC50) examination, colony formation assays and flow cytometry assays were conducted to examine the role of TNFAIP2 in vitro, while xenograft models in nude mice and 4-nitroquinoline N-oxide (4NQO)-induced HNSCC models in C57BL/6 mice were adopted to verify the effect of TNFAIP2 in vivo. Gene set enrichment analysis (GSEA) and coimmunoprecipitation coupled with mass spectrometry (Co-IP/MS) were performed to determine the mechanism by which TNFAIP2 promotes cisplatin resistance.

**Results:**

High expression of TNFAIP2 is associated with a poor prognosis, cisplatin resistance, and low reactive oxygen species (ROS) levels in HNSCC. Specifically, it protects cancer cells from cisplatin-induced apoptosis by inhibiting ROS-mediated c-JUN N-terminal kinase (JNK) phosphorylation. Mechanistically, the DLG motif contained in TNFAIP2 competes with nuclear factor-erythroid 2-related factor 2 (NRF2) by directly binding to the Kelch domain of Kelch-like ECH-associated protein 1 (KEAP1), which prevents NRF2 from undergoing ubiquitin proteasome-mediated degradation. This results in the accumulation of NRF2 and confers cisplatin resistance. Positive correlations between TNFAIP2 protein levels and NRF2 as well as its downstream target genes were validated in HNSCC specimens. Moreover, the small interfering RNA (siRNA) targeting TNFAIP2 significantly enhanced the cisplatin treatment effect in a 4NQO-induced HNSCC mouse model.

**Conclusions:**

Our results reveal the antioxidant and cisplatin resistance-regulating roles of the TNFAIP2/KEAP1/NRF2/JNK axis in HNSCC, suggesting that TNFAIP2 might be a potential target in improving the cisplatin treatment effect, particularly for patients with cisplatin resistance.

**Supplementary Information:**

The online version contains supplementary material available at 10.1186/s13046-023-02775-1.

## Background

Head and neck squamous cell carcinoma (HNSCC) exhibit high morbidity and mortality rates [1], with approximately 700,000 new cases and 380,000 deaths every year worldwide [2]. In Asian populations, the Taxol + platinum + fluorouracil (TPF) regimen is recommended as the first-line adjuvant therapy choice in advanced HNSCC, and cisplatin is the most common platinum compound used [3]. However, more than 30% of patients develop disease progression during cisplatin-based chemotherapy or within 6 months after treatment, which is called cisplatin resistance [4]. Although PD-1/PD-L1-targeted immune checkpoint therapy is recommended for those patients, the low response rate and expensive cost limit its value. The exact mechanism of cisplatin resistance in HNSCC urgently needs to be determined.

Previously, some perspectives regarding cisplatin resistance have been proposed: these include elevated cisplatin uptake efficiency [5], enhanced nucleotide excision repair [6] or interstrand crosslink repair processes [7], and bypass activation of mitogen-activated protein kinase 1 (MAPK1, so called p38) [8] signaling pathways. However, these pathways have limited translation potential. In recent years, aberrant enhancement of the antioxidant defense system has been shown to be associated with cisplatin resistance in many cancers [9–12]. Targeting antioxidant pathways, including hypoxia-inducible factor 1-alpha (HIF1A) [13] and BTB and CNC homology 1, basic leucine zipper transcription factor 1 (BACH1) [14], was found to restore cisplatin sensitivity in an oxidative stress-dependent manner.

As a novel oncoprotein [15], tumor necrosis factor alpha-induced protein 2 (TNFAIP2) was reported to be induced by retinoic acid, human papillomavirus and Epstein‒Barr virus in acute promyelocytic leukemia, cervical cancer and nasopharyngeal carcinoma, respectively [15–17]. TNFAIP2 could also be activated by amentoflavone and exhibited a protective effect against radiation-induced oxidative stress [18]. More recently, TNFAIP2 upregulation was proven to be associated with cisplatin resistance in non-small cell lung cancer and urothelial cancer [19, 20]. Nonetheless, its exact role in HNSCC as well as the underlying mechanism by which it promotes cisplatin resistance are still unclear.

In this study, we found that high TNFAIP2 expression was associated with TPF chemotherapy failure in HNSCC. Specifically, TNFAIP2 promoted cisplatin treatment resistance by inhibiting reactive oxygen species (ROS)/JNK pathway-mediated apoptosis. Transcriptomic analyses showed that TNFAIP2 exerted its antioxidant role by sustaining nuclear factor-erythroid 2-related factor 2 (NRF2, also known as NFE2L2) signaling. Additional proteomic evidence demonstrated that TNFAIP2 interacts with the Kelch domain of KEAP1 via its DLG motif, which protects NRF2 from ubiquitin proteasome-mediated degradation. The above results were validated in human specimens and the 4-nitroquinoline N-oxide (4NQO)-induced HNSCC mouse model. Our results suggest that TNFAIP2 might be a therapeutic target in improving the cisplatin treatment effect of HNSCC.

## Methods

### Human subjects and samples

Paraffin-embedded specimens from 178 HNSCC patients and 20 HNSCC patients who received TPF chemotherapy alone were obtained from the Affiliated Hospital of Stomatology, Nanjing Medical University. TPF resistance was defined as progression during platinum-based chemotherapy and within 6 months after treatment. The specimens used for gene, protein and antioxidative activity analyses were stored in liquid nitrogen immediately ex vivo. Our research was approved by the Ethics and Research Committee of Nanjing Medical University (No. 2022 − 230). Informed consent was obtained from all patients.

### Mouse and tumor models

Mice purchased from Beijing Vital River Laboratory were housed under standard specific-pathogen-free (SPF) conditions. Twenty 6-week-old male BALB/c nude mice were randomly divided into four groups (n = 5 mouse/group), and the appropriate cells (approximately 2 × 10^6^ FADU cells transfected with vector or Flag-TNFAIP2) were inoculated subcutaneously into the right flanks to create the xenograft model. N, N-Dimethylformamide (DMF, MCE, HY-Y0345) and cisplatin (APExBIO, A821, 5 mg/kg) were injected into the caudal vein every four days. Tumor volume was observed every three days and calculated by the standard formula: 0.54 × Length × Width^2^. After four weeks, tumors were collected for weighing and immunohistochemistry (IHC) examination.

The induced HNSCC model was constructed in 6-week-old C57BL/6 mice. Briefly, drinking water containing 50 mg/ml 4NQO (Sigma, N8141-5G) was consecutively administered for 16 weeks, followed by normal drinking water for 6 weeks. Subsequently, tumor burden mice were randomly divided into 4 groups (N = 7/group) and given (1) si-control; (2) cisplatin (5 mg/kg caudal vein injection every four days); (3) si-Tnfaip2 (5 nM tumor local injection every four days) and (4) cisplatin + si-Tnfaip2. After 4 weeks of treatment, all mice were sacrificed to evaluate the lesion areas. Dissected tumor tissues were fixed in 10% formalin overnight and paraffin-embedded. Every block was cut into 4 mm thick sections for hematoxylin-eosin (HE) and IHC staining. Tumor invasiveness was classified according to the following criteria: epithelial dysplasia appearance (G1); distinct invasion into the superficial portion of the muscle layer, basement membrane unclear (G2); and extensive invasion into the deep layer of muscle and loss of the basement membrane (G3). To knock down Tnfaip2 in vivo, RNase- and cholesterol-modified (2’OMe + 5’Chol) siRNA was synthesized by RiboBio (Guangzhou, China). This study was conducted in accordance with the Declaration of Helsinki (as revised in 2013).

### Cell culture and transfection

The cell lines used in this study were purchased from the American Type Culture Collection (ATCC) and cultured in an atmosphere of 95% air and 5% CO2 in Dulbecco’s modified Eagle’s medium (DMEM; Gibco) supplemented with 10% fetal bovine serum (FBS; Cellmax). All cell lines tested mycoplasma contamination negative. All small interfering RNAs (siRNAs) and wild type, truncated or mutant plasmids were constructed by GenePharma (Shanghai, China), Hanbio Biotechnology Co., Ltd. (Shanghai, China) and Ribobio (Guangzhou, China), as shown in Tables S3 and S4. All transfection experiments were conducted by using ExFect Transfection Reagent (Vazyme) following the manufacturer’s instructions.

### Western blotting

Cell and tissue lysates were prepared using RIPA buffer (Beyotime, P0013B), followed by ultrasonic cell disruption for 5 s at 20 Hz on ice. Specific antibodies were adopted against the target proteins, as shown in Table S5. The signaling intensity was detected by chemiluminescent substrate (Tanon, 180–5001). The amounts of protein were quantified by ImageJ software relative to GAPDH.

### Coimmunoprecipitation coupled with mass spectrometry (Co-IP/MS)

To detect the protein interaction with TNFAIP2, FADU cells were lysed by IP lysis buffer (Beyotime, P0013) followed by centrifugation at 120,000 r/min for 30 min at 4 °C for purification. Then, the supernatants were incubated with anti-TNFAIP2 (Santa Cruz, sc-28,318) or IgG (Proteintech, SA00001-2) plus Protein A/G β PLUS Agarose beads (Santa Cruz, sc-2003) at 4 °C overnight. Then, the reaction mixtures were harvested and centrifuged at 3000 × g for 5 min at 4 °C with IP lysis buffer and washed at least three times. The proteins interacting with TNFAIP2 were subjected to SDS‒PAGE and visualized by silver staining. With clearly visible bands appearing, liquid chromatography tandem mass spectrometry (LC‒MS) was performed on a Q Exactive mass spectrometer that was coupled to Easy nLC (Thermo Fisher Scientific) for protein identification. The MS data were analyzed using MaxQuant software version 1.3.0.5. MS data were searched against UniProt_homo_202249_20211008 (202,249 total entries, downloaded 2021/10/8). The specific conditions for silver staining and LC‒MS as well as database searching parameters are displayed in Additional file 3.

### Real-time quantitative polymerase chain reaction (RT‒qPCR)

TRIzol Reagent (Vazyme, R401-01) was used to extract total RNA from HNSCC cells or fresh-frozen tumor specimens according to the manufacturer’s instructions. cDNA was generated using equal amounts of RNA and 5× HiScript II qRT SuperMix (Vazyme, R222-01-AB). RT‒qPCR was performed using 2× ChamQ Universal SYBR qPCR Master Mix (Vazyme, Q711-02-AA) and an ABI7900 instrument (Applied Biosystems), with the 2^−∆∆Ct^ method adopted to calculate relative gene expression. GAPDH was used to normalize the data, and the results are presented as relative mRNA levels. The primer sequences used in this study are listed in Table S6.

### Cell viability and chemoresistance assay analysis

Cell Counting Kit-8 (CCK-8, APExBIO, K1018) was used to detect cell viability. Briefly, CCK-8 solution at a 10% concentration in DMEM was added to a 96-well plate. After incubation for 3 h at 37 °C, the optical density (OD) value was measured using a spectrometer at 450 nm. Each well was read independently three times.

### Colony formation assays

HNSCC cells were seeded in 6-well plates at a density of 2000 cells/well. The colonies propagated in culture and were visible (more than 50 cells) at day 10. Then, the colonies were fixed with 4% paraformaldehyde (Biosharp, BL539A) and stained with crystal violet (Beyotime, C0121), and the number of colonies in each well was determined using an optical microscope.

### Apoptosis assay

After the indicated treatments, cells were collected carefully in 100 µL phosphate buffer saline (PBS) and stained with 5 µL PI (Vazyme Biotech) and 5 µL FITC-conjugated Annexin V for 10 min at room temperature in the dark. Afterward, samples were run on a flow cytometer (BD FACSVerse, USA), and the data were analyzed by Flow Jo software 10.6.2. All the cells were gated with at least 20,000 cells collected for each sample analysis.

### ROS and antioxidant capacity detection

Transfected cells were incubated with 5 µM MitoSOX™ Green mitochondrial superoxide indicator (Thermo Fisher Scientific, M36006) for 10 min at 37 °C protected from light, followed by three washes with warm buffer. Afterward, MitoSOX™ fluorescence signals were detected by flow cytometry (BD FACSVerse, USA). A total of 10,000 cells were analyzed for each sample, and the FITC channel signal was plotted using Flow Jo software 10.6.2.

The glutathione to oxidized glutathione (GSH/GSSG) ratio was calculated using a GSH/GSSG detection assay (Abcam, ab205811) according to the manufacturer’s instructions. Superoxide dismutase (SOD) activity was evaluated by an SOD assay kit (Jiancheng, A001-3-2) according to the manufacturers’ instructions.

### IHC staining

After standard procedures, including deparaffinization, dehydration and antigen retrieval, tissue sections were incubated with primary and secondary antibodies successively. The corresponding antibodies are listed in Table S5. All stained slides were scanned by whole slide imaging (WSI) (Servicebio, Wuhan, China), and we read the slides using CaseViewer software. Two independent pathological investigators were blinded to evaluate the straining score. We divided four categories for the scope of stained-positive cells: 0 to 1 (0-25%), 1 to 2 (26-50%), 2 to 3 (51-75%), and 3 to 4 (76-100%). The following criteria were adopted to evaluate staining intensity: 0, no appreciable staining; 1, weak intensity (light yellow); 2, moderate intensity (yellow‒brown); and 3, strong intensity (brown). The staining index was calculated as the scope of stained-positive cells score × the staining intensity score. The expression of the protein was divided into high and low levels according to their mean scores.

### TUNEL staining

After deparaffinization and dehydration, the paraffin-embedded sections were incubated with protease K for 20 min in drop balancing buffer for 10–30 min at room temperature. Then, TdT-incubate buffer was added at 37 °C for 60 min, and the phosphate buffer was washed. The DAPI solution was redyed in a dark environment, and the phosphate buffer was washed. The results were immediately observed under a fluorescence microscope using standard fluorescence green fluorescence at 520 ± 20 nm for apoptotic cells and blue fluorescence at 460 nm for the total number of cells. Apoptotic cells were counted by ImageJ v1.53c software. Percentage of apoptosis = apoptotic cells/total number of cells ×100%.

### Dual-luciferase reporter gene

Antioxidant response elements (AREs) (5’-GTGACAAAGCAATCCCGTGACAAAGCAATCCCGTGACAAAGCAATA-3’) were cloned into the pGL3-basic luciferase reporter plasmid. FADU and CAL33 cells were seeded in 24-well plates (2.5 × 10^4^ cells per well) in triplicate and incubated for 24 h. Then, cells with different treatments were transfected with 200 ng ARE luciferase reporter plasmids using ExFect Transfection Reagent (Vazyme). The same amount of Renilla was included in each well and used to standardize transfection efficiency. Then, the cells were cultured in 10% FBS medium for 24 h. The Dual-Luciferase Reporter Assay kit (Beyotime, RG088S) was used to measure firefly and Renilla signals at 48 h post transfection.

### Statistical analysis

All experiments were performed at least three times, and data are represented as the means ± SEMs. Survival analysis was conducted using the Kaplan‒Meier method and Cox proportional hazard model. Comparisons were performed using Student’s *t* test for two groups and ANOVA for multiple groups. Pearson’s correlation coefficient was calculated to evaluate the correlation between gene expression, and a normal distribution was assumed. GraphPad Prism 8 and *R* studio software were used for statistical analysis. A two-sided *P* value < 0.05 was considered to indicate statistical significance. The level of significance is denoted as *P < 0.05, **P < 0.01, and ***P < 0.001.

## Results

### Patients with high TNFAIP2 expression tend to experience cisplatin treatment failure in HNSCC

**Fig. 1:**
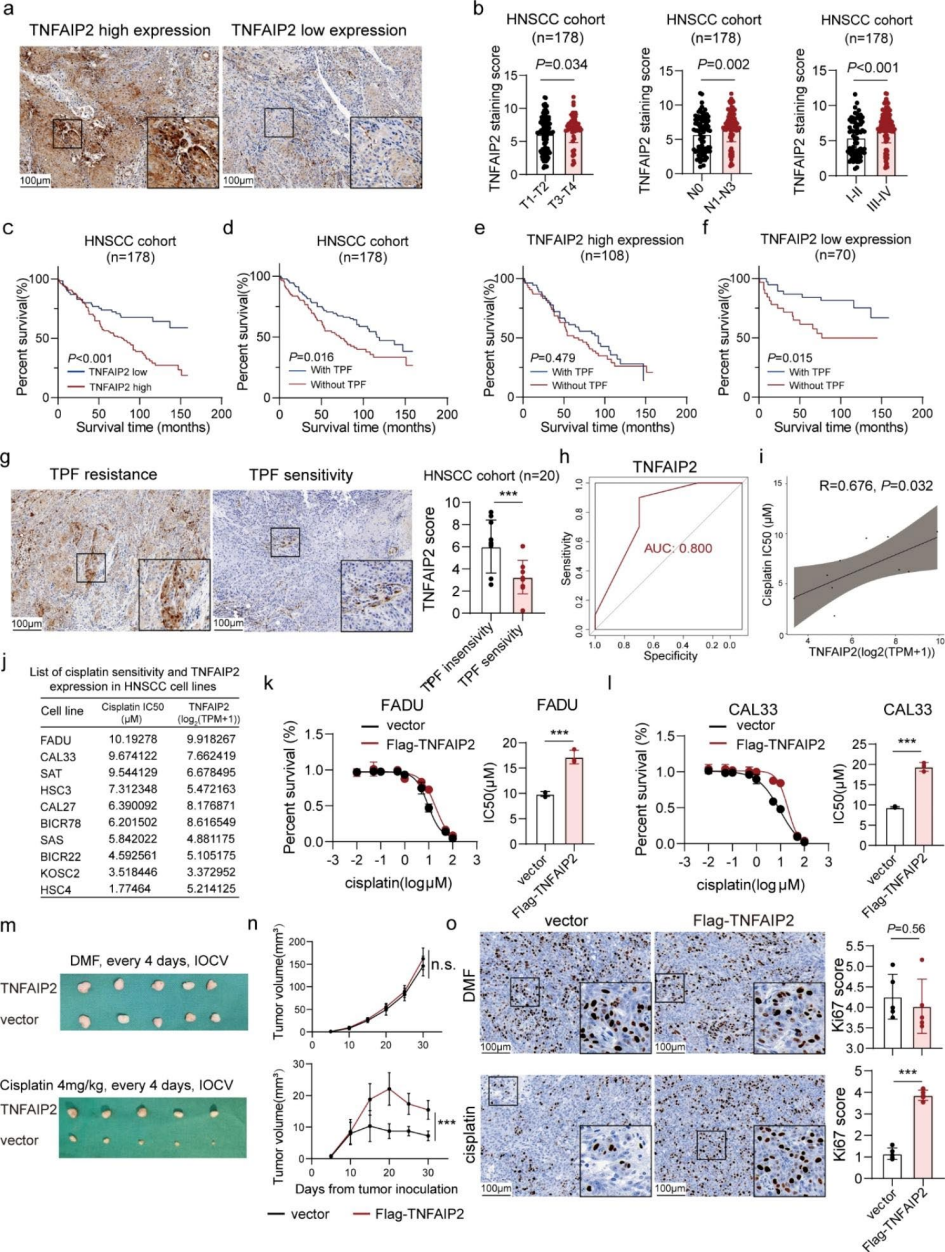
Patients with HNSCC with high TNFAIP2 expression tend to experience cisplatin treatment failure. (**a**) IHC staining for TNFAIP2 in the human HNSCC cohort. (**b**) TNFAIP2 protein expression in different T, N and clinical stages. (**c**) Kaplan‒Meier survival curve of HNSCC patients with low versus high TNFAIP2 expression. (**d-f**) Kaplan‒Meier survival curve of HNSCC patients with or without TPF chemotherapy in the whole group (**d**), high TNFAIP2 expression group (**e**) and low TNFAIP2 expression group (**f**). (**g**) IHC staining for TNFAIP2 in TPF chemotherapy-resistant and TPF chemotherapy-sensitive HNSCC patients. (**h**) Accuracy of TNFAIP2 level in predicting TPF chemotherapy response of HNSCC. (**i-j**) Correlation between TNFAIP2 mRNA expression and cisplatin IC50 in 10 HNSCC cell lines. (**k-l**) Cisplatin IC50 evaluations in TNFAIP2-overexpressing FADU (k) and CAL33 (l). (**m**) Xenograft images in nude mice after the indicated treatments. (**n**) Tumor growth curves of each group after the indicated treatments. (**o**) IHC staining for Ki67 in xenografts of each group. Scale bars, 100 μm. Data are presented as the mean ± SEM. n.s., not significant; *** *P* < 0.001. TPF, Taxol + platinum + fluorouracil; AUC, area under the curve; DMF, N, N-dimethylformamide; IOCV, tail vein injection

To explore the expression and potential role of TNFAIP2 in HNSCC, we first referred to The Cancer Genome Atlas (TCGA) and Gene Expression Omnibus (GEO) databases. The results showed that TNFAIP2 mRNA was significantly upregulated in HNSCC tissues compared to normal tissues (*P* = 0.008) (Figure S1a), while no obvious correlation with survival was seen from the TCGA (*P* = 0.690) and GEO (GSE65858, *P* = 0.950) (Figure S1b and c). Then, we performed IHC staining based on 178 HNSCC patients [105 (59.0%) males, 92 (51.7%) received TPF chemotherapy] (Table S1). We found that TNFAIP2 was upregulated in advanced-stage patients, suggesting its tumor-promoting role (Fig. 1a and b). High TNFAIP2 expression indicated poor overall survival (Fig. 1c) and was proven to be an independent risk factor (HR: 1.844; 95% CI: 1.139–2.983; *P* = 0.013) (Table S2). Interestingly, the TNFAIP2 expression level was undifferentiated in the TPF and without TPF groups (*P* = 0.845) (Figure S1d), we observed that TPF chemotherapy achieved superior treatment outcomes in the TNFAIP2 low expression subgroup vs. the TNFAIP2 high expression subgroup (Fig. 1d-f). This implied that TNFAIP2 might be associated with TPF treatment resistance in HNSCC. In another cohort (n = 20) that HNSCC patients received TPF treatment alone, TNFAIP2 was significantly upregulated in TPF treatment-resistant patients (Fig. 1g). The expression level effectively distinguished suitable candidates for TPF treatment [area under the curve (AUC) = 0.800] (Fig. 1h).

We further examined correlations between TNFAIP2 mRNA levels and half-maximal inhibitory concentrations (IC50s) for Taxol, cisplatin and 5-fluorouracil in 10 HNSCC cell lines. The results revealed positive correlations between the cisplatin IC50 and TNFAIP2 expression (R = 0.676, *P* = 0.032; Fig. 1i and j and S1e). Based on this information, we selected FADU and CAL33 for further explorations (Figure S1f). Cell viability and colony formation experiments revealed that TNFAIP2 had little impact on cell proliferation. However, its overexpression reduced cisplatin toxicity (Figure S1g-i). Moreover, the IC50 results verified that TNFAIP2 overexpression increased cisplatin resistance, while its knockdown decreased cisplatin resistance (Fig. 1k and l and S1j and k). Subsequently, we established xenograft tumor models by subcutaneously inoculating FADU into BALB/c nude mice with the indicated treatment (Fig. 1m). The results showed that TNFAIP2 overexpression had no influence on either tumor weight or volume, while it significantly diminished the treatment effect of cisplatin (Fig. 1n and S1l). Ki67 staining verified this effect in vivo (Fig. 1o). Collectively, the above results revealed the adverse role of TNFAIP2 in cisplatin treatment of HNSCC.

### TNFAIP2 protects HNSCC cells from cisplatin-induced apoptosis by inhibiting ROS/JNK signaling

**Fig. 2:**
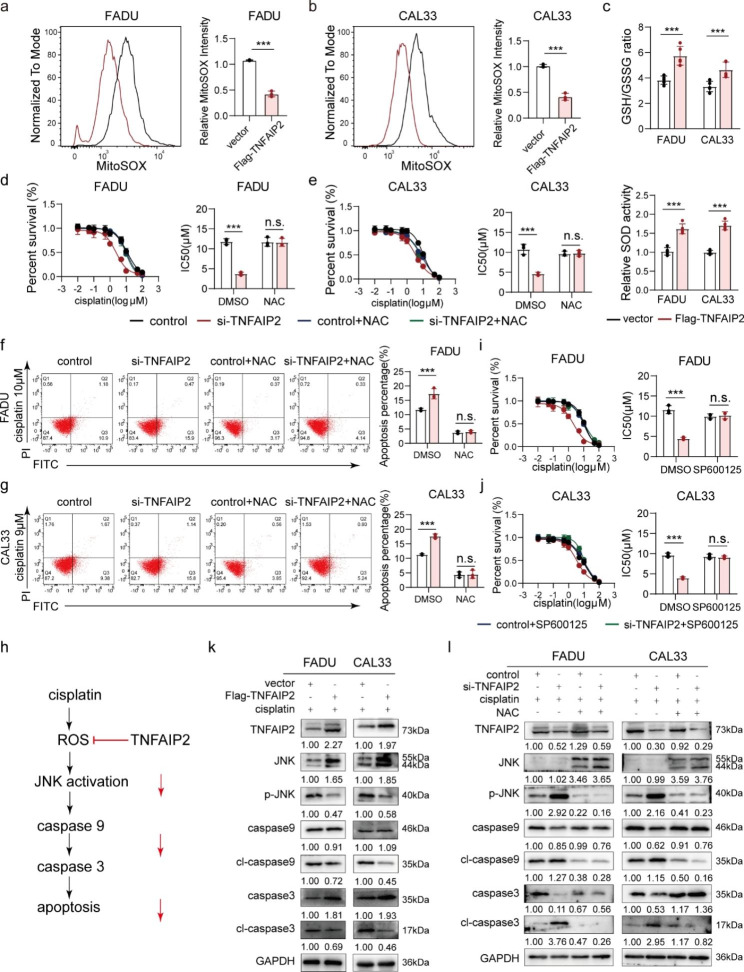
TNFAIP2 protects HNSCC cells from cisplatin-induced apoptosis by inhibiting ROS/JNK signaling. (**a-b**) Flow cytometry analyses of ROS in TNFAIP2-overexpressing FADU (**a**) and CAL33 cells (**b**). (**c**) Analyses of GSH/GSSG and SOD in TNFAIP2-overexpressing HNSCC cell lines. (**d-e**) Cisplatin IC50 evaluations in TNFAIP2-knockdown FADU (**d**) and CAL33 (**e**) cells with or without NAC (4 mmol/L, 2 **h**). (**f-g**) Flow cytometry analyses of cisplatin-induced apoptosis in TNFAIP2-knockdown FADU (**f**) and CAL33 (**g**) cells with or without NAC (4 mmol/L, 2 h). (**h**) Schematic diagram of ROS/JNK signaling in cisplatin-induced apoptosis. (**i-j**) Cisplatin IC50 evaluations in TNFAIP2-knockdown FADU (i) and CAL33 (j) cells with or without the JNK pathway inhibitor SP600125 (20 µmol/L, 2 h). (**k**) Western blot analysis of the molecular markers for JNK and apoptosis signaling induced by cisplatin in vector- or TNFAIP2-overexpressing HNSCC cell lines. (**l**) Western blot analysis of the molecular markers for JNK and apoptosis signaling induced by cisplatin in control or TNFAIP2 knockdown HNSCC cell lines with or without NAC (4 mmol/L, 2 h). Data are presented as the mean ± SEM. n.s., not significant; ** *P* < 0.01; *** *P* < 0.001. NAC, N-acetylcysteine

According to previous reports, malignant tumors escape from cisplatin-induced apoptosis through multiple mechanisms. However, TNFAIP2 overexpression did not impact the cell cycle distribution (Figure S2a) [21] or the expression of a set of cisplatin resistance-associated genes (Figure S2b). Interestingly, we found that the ROS level was reduced when TNFAIP2 was overexpressed (Fig. 2a and b). Moreover, the GSH/GSSG ratio as well as the SOD activity also increased, suggesting alleviation of oxidative stress (Fig. 2c). When TNFAIP2 was knocked down, the opposite results were obtained (Figure S2c-e). As cisplatin-induced apoptosis was also reduced when TNFAIP2 was overexpressed (Figure S2f and g), we speculated that TNFAIP2 conferred cisplatin resistance by accelerating ROS elimination. As expected, the ROS scavenger N-acetylcysteine (NAC) restored cisplatin resistance induced by TNFAIP2 knockdown (Fig. 2d-g).

Overwhelming evidence indicates that excessive ROS induce apoptosis by persistently activating the JNK pathway [22–24]. We hypothesized that TNFAIP2 protected cancer cells from cisplatin cytotoxicity in a ROS/JNK pathway-inhibited manner (Fig. 2h). As a result, the JNK inhibitor (SP600125) reversed the increase in apoptosis rate and cisplatin sensitivity induced by TNFAIP2 knockdown (Fig. 2i and j and S2h and i). Western blot analysis showed that cisplatin-induced phosphorylation of JNK and activation of caspase 9 and caspase 3 were consistently inhibited when TNFAIP2 was overexpressed (Fig. 2k). NAC reversed signaling enhancement induced by TNFAIP2 knockdown (Fig. 2l), indicating that TNFAIP2 protected HNSCC cells from cisplatin-induced apoptosis by inhibiting the ROS/JNK pathway.

### TNFAIP2 stabilizes the NRF2 protein by inhibiting its ubiquitination and degradation

**Fig. 3:**
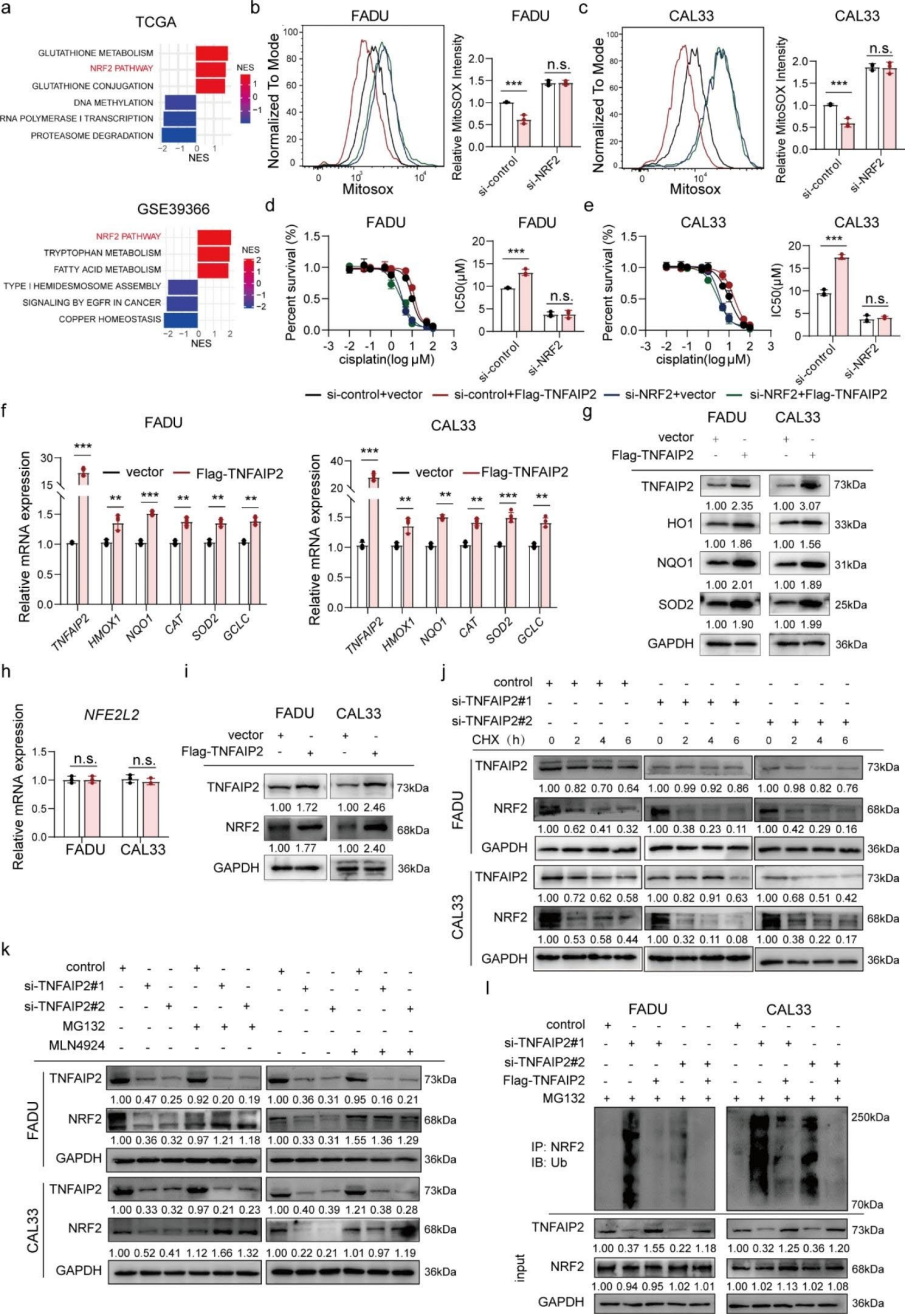
TNFAIP2 stabilizes the NRF2 protein by inhibiting its ubiquitination degradation. (**a**) GSEA of signaling pathways significantly correlated with TNFAIP2 in TCGA-HNSCC and GSE39366. (b-c) Flow cytometry analyses of ROS in TNFAIP2-overexpressing FADU (**b**) and CAL33 (**c**) cells with or without NRF2 knockdown. (**d-e**) Cisplatin IC50 evaluations in TNFAIP2-overexpressing FADU (d) and CAL33 (e) cells with or without NRF2 knockdown. (**f-g**) The mRNA (f) and protein (g) levels of NRF2 target genes in TNFAIP2-overexpressing HNSCC cell lines. (**h-i**) The mRNA (h) and protein (i) levels of NRF2 in TNFAIP2-overexpressing HNSCC cell lines. (**j**) The NRF2 protein level in TNFAIP2 knockdown HNSCC cell lines treated with CHX (50 µg/ml) at the corresponding time points. (**k**) The NRF2 protein level in TNFAIP2 knockdown HNSCC cell lines treated with or without MG132 (10 µM, 4 h) or MLN4924 (2 µM, 1 h). (**l**) Co-IP analyses of NRF2 ubiquitination in HNSCC cell lines with the indicated treatments treated with MG132 (10 µM, 4 h). Data are presented as the mean ± SEM. n.s., not significant; ** *P* < 0.01; *** *P* < 0.001. GSEA, gene set enrichment analysis

Further enrichment analysis based on TCGA and GEO (GSE39366) data showed that NRF2 signaling was prominently upregulated in tissues with high TNFAIP2 expression (Fig. 3a). We concluded that the antioxidant effect of TNFAIP2 was mediated by NRF2. As expected, when NRF2 was knocked down, TNFAIP2 overexpression did not impact the ROS level (Fig. 3b and c), while the cisplatin resistance-promoting ability also vanished (Fig. 3d and e). To monitor NRF2 transcriptional activity, we constructed an ARE-containing dual-luciferase reporter system. Obviously, the luciferase activity increased when TNFAIP2 was overexpressed (Figure S3a), while it decreased after TNFAIP2 knockdown (Figure S3b). Moreover, a set of NRF2 target genes, including heme oxygenase 1 (HO1), NADPH quinone oxidoreductase 1 (NQO1), glutamate-cysteine ligase catalytic subunit (GCLC), catalase (CAT) and superoxide dismutase 2 (SOD2), were altered along with TNFAIP2 at both the mRNA and protein levels (Fig. 3f and g and S3c and d). These results demonstrated that the antioxidant and cisplatin resistance functions of TNFAIP2 were mediated by NRF2.

Then, we found that TNFAIP2 impacted the protein level rather than the mRNA level of NRF2 (Fig. 3 h and i and S3e and f), suggesting that the regulatory effect occurred in the posttranscriptional stage. After treatment with cycloheximide (CHX), NRF2 degraded more rapidly in the TNFAIP2 knockdown group (Fig. 3j), indicating that TNFAIP2 inhibited NRF2 protein degradation but does not facilitate its synthesis. Subsequently, we found that the proteasome inhibitor MG132 or the neddylation inhibitor MLN4924 blocked NRF2 degradation when TNFAIP2 was knocked down (Fig. 3k). Furthermore, coimmunoprecipitation (Co-IP) assays showed that ubiquitin-conjugated NRF2 increased when TNFAIP2 was knocked down, which could be restored by TNFAIP2 overexpression (Fig. 3l). In summary, TNFAIP2 exhibits antioxidant and cisplatin resistance functions by promoting the protein stabilization of NRF2.

### TNFAIP2 interacts with KEAP1 to stabilize NRF2

**Fig. 4:**
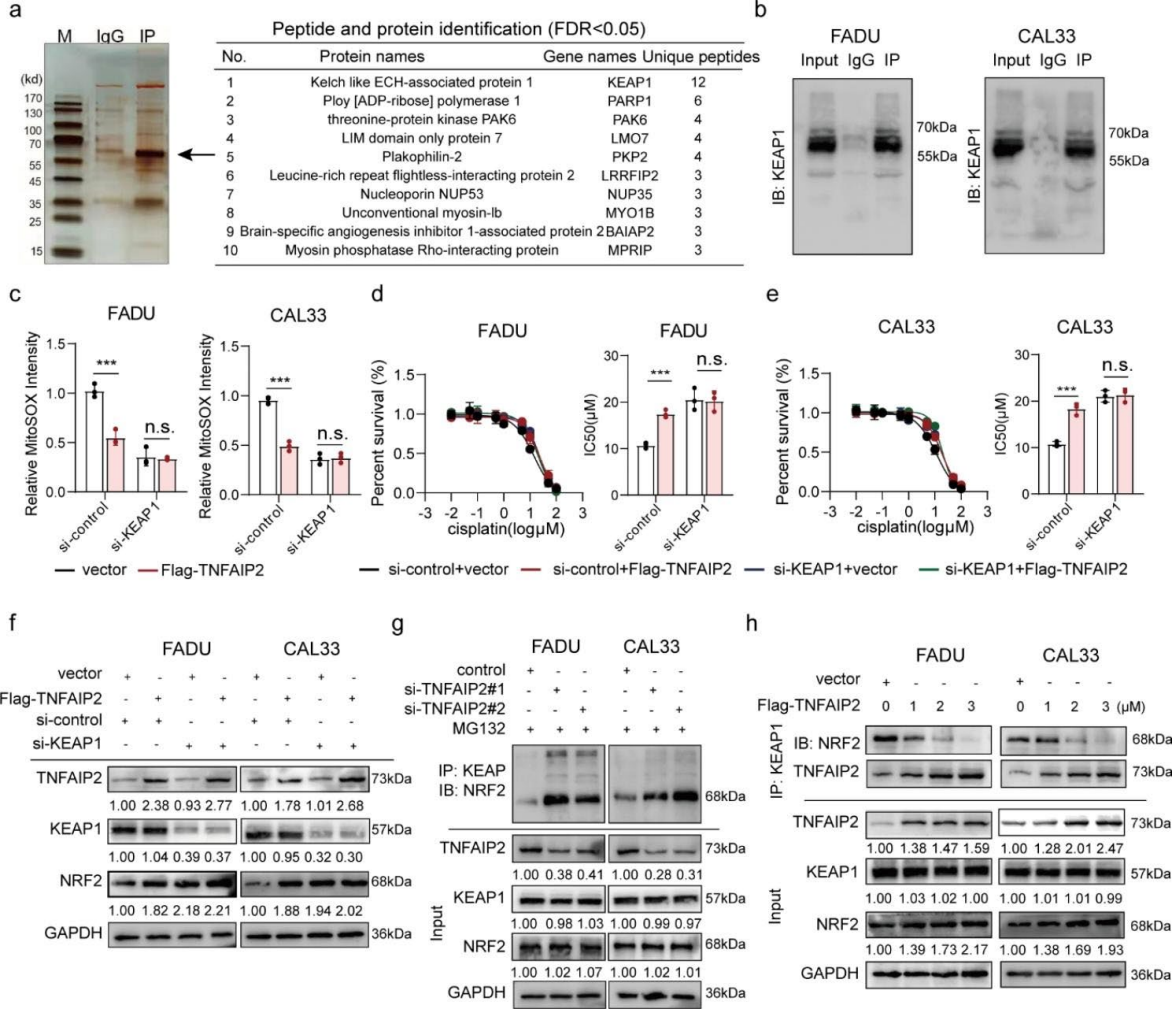
TNFAIP2 interacting with KEAP1 results in NRF2 stabilization. (**a**) IP/MS assay of TNFAIP2-interacting proteins. (**b**) Co-IP assay of the TNFAIP2 and KEAP1 interaction in HNSCC cell lines. (**c**) Flow cytometry analyses of ROS in TNFAIP2-overexpressing HNSCC cell lines with or without KEAP1 knockdown. (**d-e**) Cisplatin IC50 evaluations in TNFAIP2-overexpressing FADU (d) and CAL33 (e) cells with or without KEAP1 knockdown. (**f**) Western blot analysis of NRF2 in TNFAIP2-overexpressing HNSCC cell lines with or without KEAP1 knockdown. (**g**) Co-IP assay of the amount of NRF2 immunoprecipitated by KEAP1 in TNFAIP2 knockdown HNSCC cell lines treated with MG132 (10 µM, 4 h). (**h**) Co-IP assay of the amount of NRF2 immunoprecipitated by KEAP1 in TNFAIP2-overexpressing HNSCC cell lines. Data are presented as the mean ± SEM. n.s., not significant; *** *P* < 0.001. IP/MS, immunoprecipitation coupled with mass spectrometry

To determine the mechanisms by which TNFAIP2 stabilizes NRF2, we performed high-throughput screening using Co-IP/MS. The results showed an extensive binding relationship between TNFAIP2 and KEAP1 (Fig. 4a and S4a), which was validated by Co-IP (Fig. 4b). We speculated that KEAP1 might mediate the oxidative stress inhibition and cisplatin resistance-promoting effects of TNFAIP2. To verify this, we knocked down KEAP1 and found TNFAIP2 overexpression did not lead to ROS reduction (Fig. 4c), IC50 elevation (Fig. 4d and e), NRF2 protein accumulation (Fig. 4f) or luciferase activity enhancement (Figure S4b).

Further experiments showed that TNFAIP2 knockdown had no perceptible impact on either the mRNA or protein levels of KEAP1 and sequestosome 1 (SQSTM1) (the coding gene of p62) (Figure S4c and d). The Co-IP assays did not reveal an interaction between TNFAIP2 and NRF2 (Figure S4e). We speculated that TNFAIP2 competitively bound to KEAP1, which prevented the ubiquitin-mediated proteasomal degradation of NRF2. As shown in Fig. 4g, Co-IP assays revealed that when TNFAIP2 was knocked down, KEAP1 bound more NRF2. Conversely, when TNFAIP2 was overexpressed in a concentration gradient, the amount of NRF2 precipitated by KEAP1 decreased gradually (Fig. 4h).

### The DLG motif and kelch domain mediate TNFAIP2 interaction with KEAP1

**Fig. 5:**
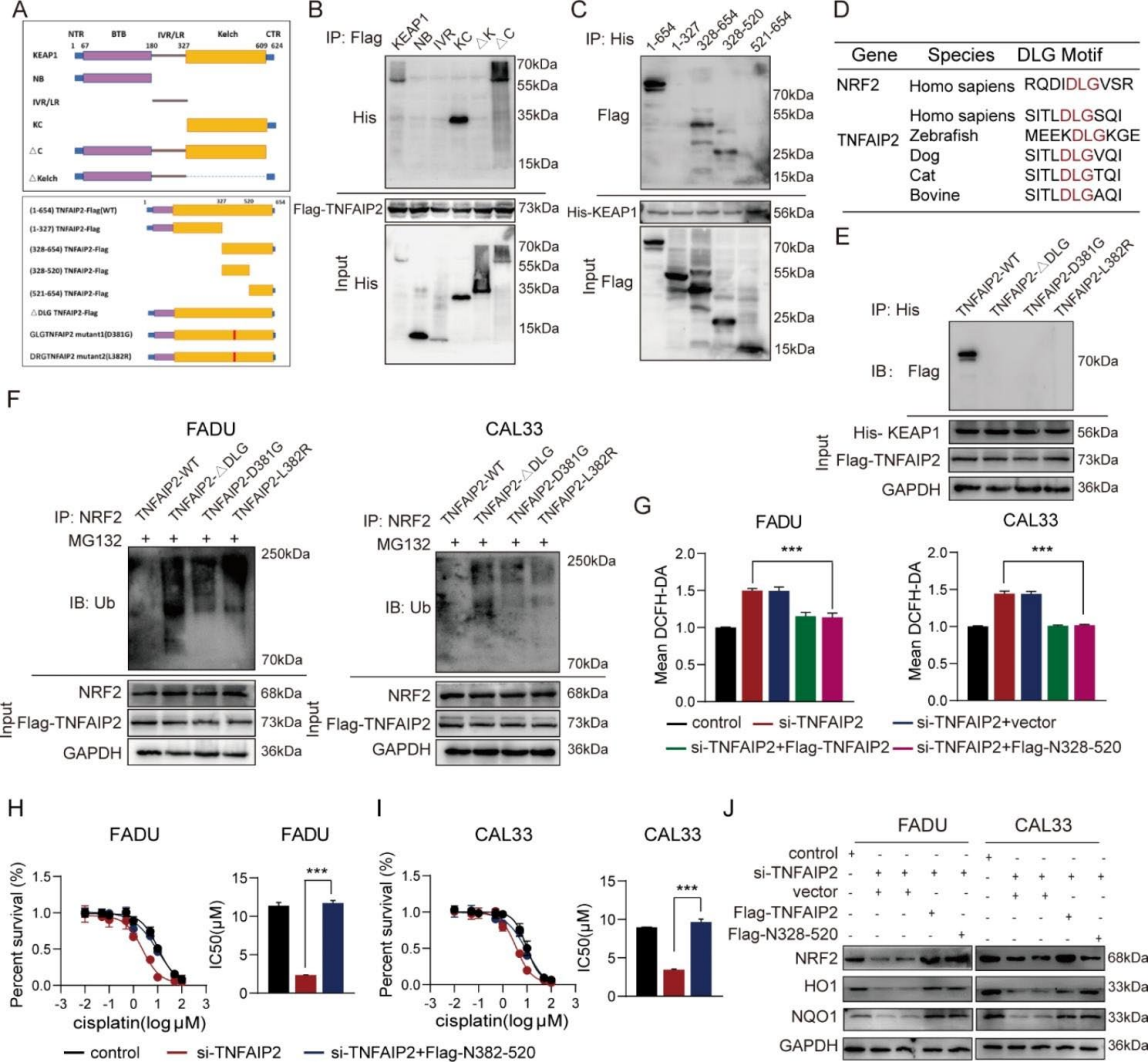
The DLG motif and Kelch domain mediate TNFAIP2 interaction with KEAP1. (**a**) Schematic diagram of wild-type and truncated or mutant TNFAIP2 and KEAP1. (**b**) Co-IP assay of KEAP1 fragments interacting with TNFAIP2 in 293T cells. (**c**) Co-IP assay of TNFAIP2 fragments interacting with KEAP1 in 293T cells. (**d**) The putative motif mediates the interaction of TNFAIP2 with KEAP1 across different species. (**e**) Co-IP assay of the DLG motif within TNFAIP2 interacting with KEAP1 in 293T cells. (**f**) Co-IP analyses of NRF2 ubiquitination in HNSCC cell lines transfected with the wild-type or mutant DLG motif treated with MG132 (10 µM, 4 h). (**g**) Flow cytometry analyses of ROS in TNFAIP2 knockdown HNSCC cell lines rescued by expression of wild-type TNFAIP2 or the N328-520 fragment. (**h-i**) Cisplatin IC50 evaluations in TNFAIP2 knockdown FADU (h) and CAL33 (i) rescued by expression of wild-type TNFAIP2 or the N328-520 fragment. (**j**) Western blot analysis of NRF2 and its target genes in TNFAIP2 knockdown HNSCC cell lines rescued by expression of wild-type TNFAIP2 or the N328-520 fragment. Data are presented as the mean ± SEM. *** *P* < 0.001

In this section, we constructed a series of truncated plasmids to explore the specific interaction loci between TNFAIP2 and KEAP1 (Fig. 5a). First, Flag-tagged TNFAIP2 full-length and His-tagged KEAP1 fragment plasmids were cotransfected into 293T cells. A co-IP assay showed that only the fragments containing the Kelch domain could interact with TNFAIP2 (Fig. 5b). Similarly, the full-length (N1-654) and other two fragments (N328-654 and N328-520) of TNFAIP2 were precipitated by KEAP1, which meant that TNFAIP2 interacted with KEAP1 via the N328-520 fragment (Fig. 5c). Intriguingly, we found that the DLG motif was located exactly within the N328-520 fragments of TNFAIP2, which was highly conserved across different species (Fig. 5d). According to previous studies [25–27], the combination of NRF2 with KEAP1 relies on the DLG and ETGE motifs, and this phenomenon is called the hinge and latch mechanism. Therefore, we constructed one DLG deletion (ΔDLG) and two mutant (D381G and L382R) Flag-tagged plasmids (Fig. 5a). As expected, none of the three could interact with KEAP1 (Fig. 5e). The NRF2 ubiquitination levels did not decrease in the three plasmid transfection groups compared to the group with wild-type TNFAIP2 (Fig. 5f).

Additionally, a series of rescue experiments were performed to validate the role of the DLG motif. We found that the overexpressed N328-520 fragment reversed the increased ROS level, elevated cisplatin sensitivity and decreased NRF2 abundance resulting from TNFAIP2 interference (Fig. 5g-j). Furthermore, this fragment elevated the ARE-containing luciferase activity and NRF2 target gene expression (Figure S5a and b). However, DLG-deletion TNFAIP2 transfection did not have the above effects (Figure S5c-g). These results demonstrate that the DLG motif and Kelch domain mediate TNFAIP2 interaction with KEAP1 and the development of cisplatin resistance.

#### Targeting Tnfaip2 promotes the cisplatin treatment effect in vivo

**Fig. 6:**
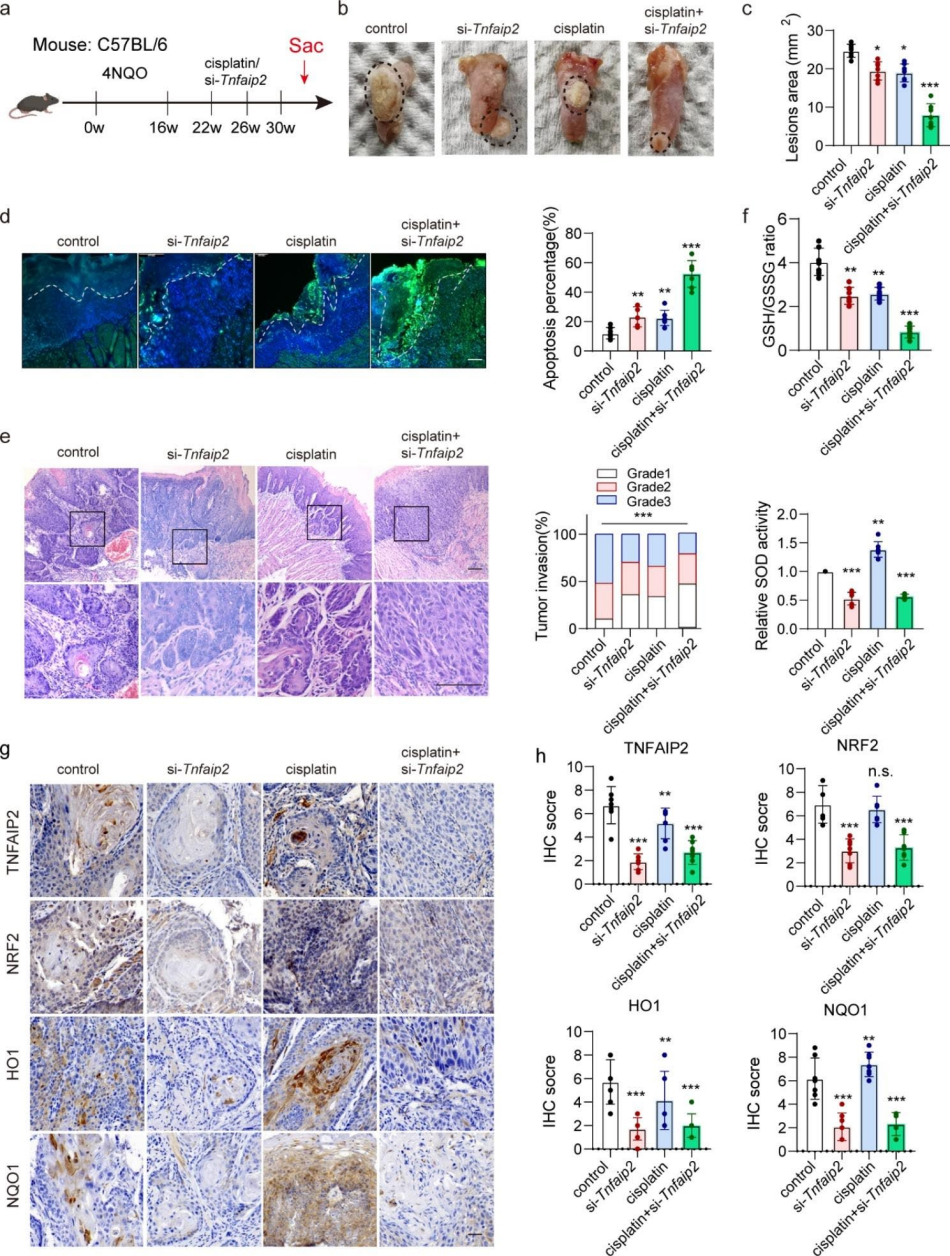
Targeting *Tnfaip2* promotes the cisplatin treatment effect in vivo. (**a**) Schematic diagram of 4NQO-induced HNSCC in C57BL/6 mice. (**b-c**) Representative visible lesion images (b) and lesion area quantification (c) in different treatment groups. (**d**) Representative TUNEL fluorescence staining images and apoptosis percentage quantification in different treatment groups. (**e**) Representative H&E staining and invasion grade quantification in different treatment groups. (**f**) Analyses of GSH/GSSG and SOD in different treatment groups. (**g-h**) IHC staining for TNFAIP2, NRF2, HO1 and NQO1 (g) and quantification (h) in different treatment groups. Scale bars, 100 μm. Data are presented as the mean ± SEM. n.s., not significant; * *P* < 0.05; ** *P* < 0.01; *** *P* < 0.001

To explore the promoting effect of inhibiting TNFAIP2 on cisplatin treatment, we established the 4NQO-induced HNSCC mouse model (Fig. 6a). RNase- and cholesterol-modified (2’OMe + 5’Chol) siRNA was generated and intratumorally injected to inhibit Tnfaip2 expression in vivo. Interestingly, the tumor lesion area was reduced in the Tnfaip2 knockdown group, but this effect was not significant when TNFAIP2 was overexpressed in a xenograft nude mouse model. Moreover, the combination of si-Tnfaip2 and cisplatin resulted in the most significant tumor inhibition and apoptosis induction effects (Fig. 6b-d). Meanwhile, tumor invasiveness was markedly suppressed (Fig. 6e). The GSH/GSSG ratio and SOD activity decreased consistently, suggesting aggravated intratumoral oxidative stress when Tnfaip2 was knocked down (Fig. 6f). IHC staining indicated that the expression of NRF2 and its target genes were effectively reduced at the protein level when Tnfaip2 was inhibited (Fig. 6g and h). The above results confirm that Tnfaip2-specific siRNA could improve the cisplatin treatment outcome by inhibiting NRF2 signaling in HNSCC.

### TNFAIP2 expression positively correlates with NRF2 expression in HNSCC patients

**Fig. 7:**
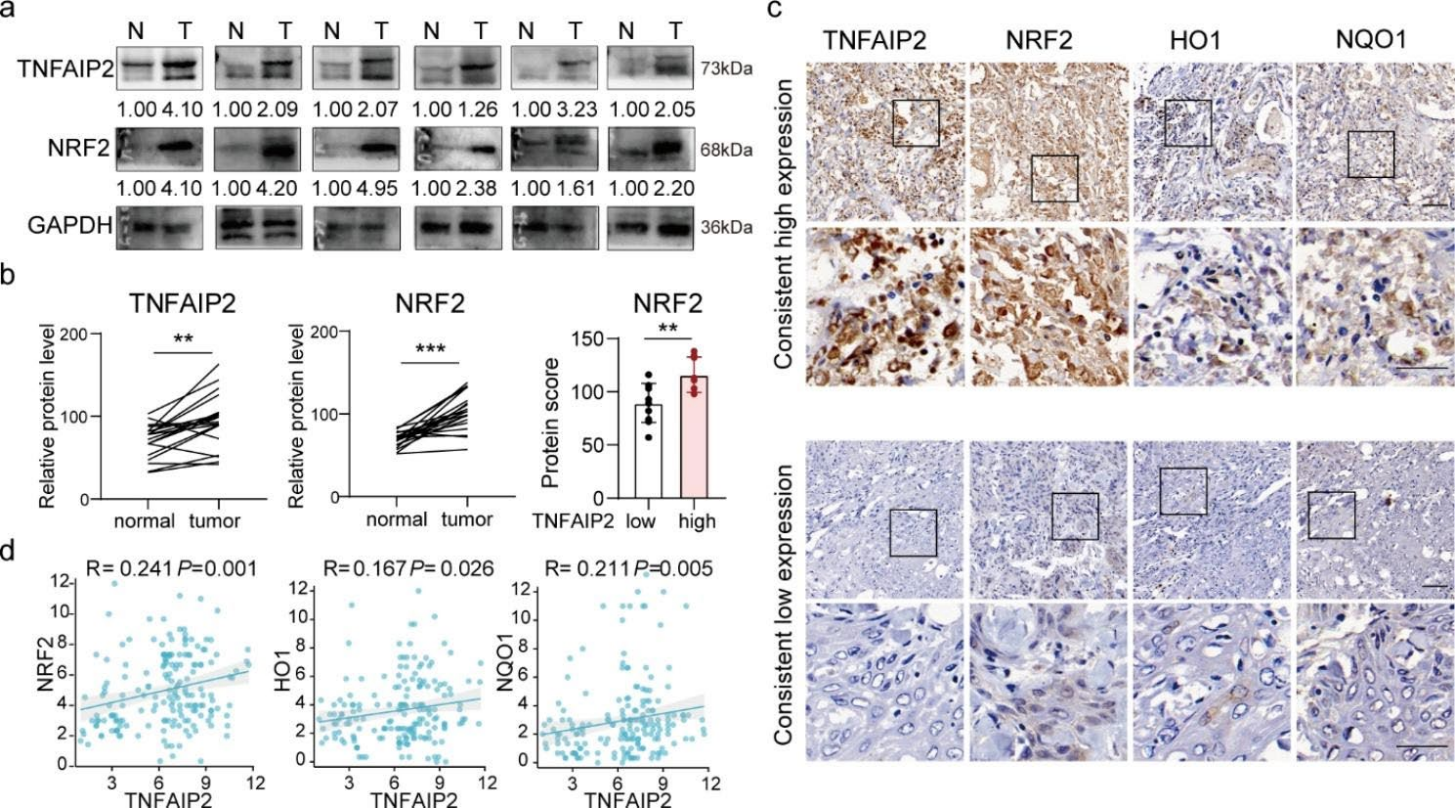
TNFAIP2 expression positively correlates with NRF2 expression in HNSCC patients. (**a-b**) Western blot analysis (a) and quantification (b) of TNFAIP2 and NRF2 in 30 pairs of HNSCC tissues (T) and normal tissues (N). (**c-d**) IHC staining (c) and correlations (d) for TNFAIP2, NRF2, HO1 and NQO1 in the human HNSCC cohort in Fig. 1. (**e**) Schematic diagram of TNFAIP2 inhibiting ROS/JNK signaling and conferring cisplatin resistance by interacting with KEAP1 and activating NRF2. Scale bars, 100 μm. Data are presented as the mean ± SEM. ** *P* < 0.01; *** *P* < 0.001

To validate the expression levels and correlations of TNFAIP2 and NRF2 in HNSCC, we first examined the expression of TNFAIP2 and NRF2 in 20 pairs of HNSCC tissues using western blotting. Compared to that in adjacent normal tissues, TNFAIP2 and NRF2 expression was higher in HNSCC tissues (Fig. 7a). In tissues with low TNFAIP2 expression, NRF2 was also downregulated, and vice versa (Fig. 7b). Additionally, we detected the expression of NRF2 and its target genes based on the cohort in Fig. 1. IHC staining showed positive correlations of TNFAIP2 with NRF2, HO1 and NQO1 (Fig. 7c and d), implying that TNFAIP2 may serve as an NRF2 signaling promoter in HNSCC.

## Discussion

Platinum-based TPF chemotherapy is widely used in the clinical treatment of HNSCC. However, approximately 30% of patients exhibit resistance to this treatment. Accumulated evidence indicates that aberrant activation of antioxidant genes is a critical obstacle in chemotherapy. Inhibition of redox-associated markers may promote the treatment effect. In this study, TNFAIP2 was found to be associated with cisplatin resistance in HNSCC. Its upregulation protects cancer cells from cisplatin-induced oxidative stress by inhibiting ROS/JNK signaling. Moreover, we found that the DLG motif contained in TNFAIP2 competes with NRF2 by interacting with the Kelch domain of KEAP1. The 4NQO-induced HNSCC mouse model proves that inhibiting TNFAIP2 is a potential approach for improving cisplatin sensitivity in HNSCC treatment, especially for those with chemotherapy resistance.

Increasing research has shown the oncogenic role of TNFAIP2 [28, 29]. Its gene polymorphisms are correlated with disease risk and a poor prognosis in gastric and cervical cancer [30, 31]. In addition, TNFAIP2 can activate ras-related C3 botulinum toxin substrate 1 and cell division cycle 42 [17, 29], which promote the proliferation, migration and cytoskeletal remodeling of tumor cells. In our cohort, HNSCC patients with TNFAIP2 protein upregulation tended to have a poor prognosis, though the transcriptome data from the TCGA and GEO did not show an obvious correlation of TNFAIP2 mRNA expression with survival. We consider this might first be due to the differences in patient data source and treatment response. Then, the transcriptome data from the TCGA and GEO are used for bulk mRNA profiling, while our IHC assessment mainly concentrate in the protein content of tumor cells. TNFAIP2 mainly exerts its functions at the protein level. Therefore, we adopt IHC to examine the prognostic value of TNFAIP2 in this study.

In non-small cell lung cancer (NSCLC) and urothelial cancer, TNFAIP2 is significantly correlated with cisplatin resistance, but the underlying mechanism needs to be explored [19, 20]. Recently, studies have indicated the aberrantly enhanced oxidative defense function of tumor cells as one of the important contributors to cisplatin treatment resistance. The regulation of intracellular oxidative stress levels can affect tumor sensitivity to cisplatin treatment. In ovarian cancer, calcium/calmodulin-dependent protein kinase II gamma (CAMK2G) facilitates adaptive redox homeostasis upon cisplatin treatment and drives cisplatin resistance by controlling the phosphorylation of ITPKB at serine 174 [11]. Similarly, 11β-hydroxy-ent-16-kaurene-15-one disrupts redox balance in NSCLC and sensitizes cisplatin (CDDP)-resistant A549 cancer cells to apoptosis and ferroptosis by targeting peroxiredoxin I/II and depleting GSH [12]. In our work, we revealed that the upregulation of TNFAIP2 drives cisplatin resistance by sustaining NRF2 signaling-mediated antioxidant activity in HNSCC. This offers a potential strategy for enhancing the cisplatin treatment effect.

NRF2 signaling is the nuclear pathway in cellular oxidative defense, and its aberrant activity is involved in multiple diseases, including cancers. In general, the dissociation of KEAP1 and NRF2 is the primary event for activating NRF2 signaling. Some posttranscriptional modifications, such as the covalent modification [32] or alkylation of KEAP1 [33], were also correlated with NRF2 accumulation. For instance, Wang et al. [34] and Jain et al. [35] reported that NESTIN and p62 could bind to KEAP1 and protect NRF2 from ubiquitination in the same way. Recently, Liu et al. [36] showed tripartite motif containing 22 (TRIM22), could inhibit the NRF2 signaling pathway by binding to and destabilizing NRF2 independent of KEAP1. Our work revealed that TNFAIP2 promotes NRF2 protein stabilization and alleviates cisplatin-induced apoptosis by interacting with KEAP1 in HNSCC. In addition, NRF2 and KEAP1 mutations can induce aberrant NRF2 signaling, which is commonly observed in lung cancer [37]. In HNSCC, there were 8.99% and 4.78% mutations for NRF2 and KEAP1, respectively, according to the TCGA. Because the cancer cell lines in the present study are wild type for both factors, further study is needed to determine whether genetic alterations impact signaling activation and clinical outcome in HNSCC.

Previous studies showed that the Kelch domain of KEAP1 could bind to the DLG and ETGE motifs, with the ETGE motif exhibiting greater affinity [38]. Oxidative and electrophilic stresses lead to the covalent modification of KEAP1 and decrease its affinity for the DLG motif of NRF2. This results in the stabilization of NRF2, accumulation of de novo-synthesized NRF2, and nuclear translocation. Therefore, the DLG motif is the main motif involved in the regulation of NRF2. Some molecules that possess DLG or ETGE motifs or that resemble DLG or ETGE motifs can disrupt the binding of KEAP1 and NRF2, which is recognized as one of the most important mechanisms by which tumor cells adapt to antioxidant activities. Ge et al. [26] reported that the DLT motif of IASPP could bind to KEAP1, showing an antioxidant function, which suggested that the function of DL* motifs was similar to that of the DLG motif. Fukutomi et al. [39] recently identified that the DLG motif is longer [DLG motif (M17–G51)] than the classical DLG motif and DIDLID element (whose lengths are based on previous assumptions). Interestingly, there are also exclusive DL* motifs within TNFAIP2, which can partly explain the higher affinity of TNFAIP2 vs. NRF2 for KEAP1. However, the specific underlying mechanisms need to be further explored.

The heterogeneity of malignancies makes it impossible to treat them with a universal approach. Some auxiliary measures may be more suitable for different tumor subtypes. Genetic therapy provides us with new insight into effectively inhibiting specific genes. Although the modified siRNA that inhibits Tnfaip2 exhibits a favorable effect in vivo, more large-scale animal experiment and advanced nanodrug delivery materials are indispensable for developing an effective strategy for targeting TNFAIP2 in the future. Notably, targeting Tnfaip2 in healthy mice (rather than xenograft tumor models) could also restrict tumor growth. We hypothesized that the locally injected siRNA downregulated TNFAIP2 indistinguishably across different cell types, which reduced the immune escape probability or increased the infiltration of cytotoxic T cells. The specific mechanism needs to be further explored.

## Conclusions

**Fig. 8:**
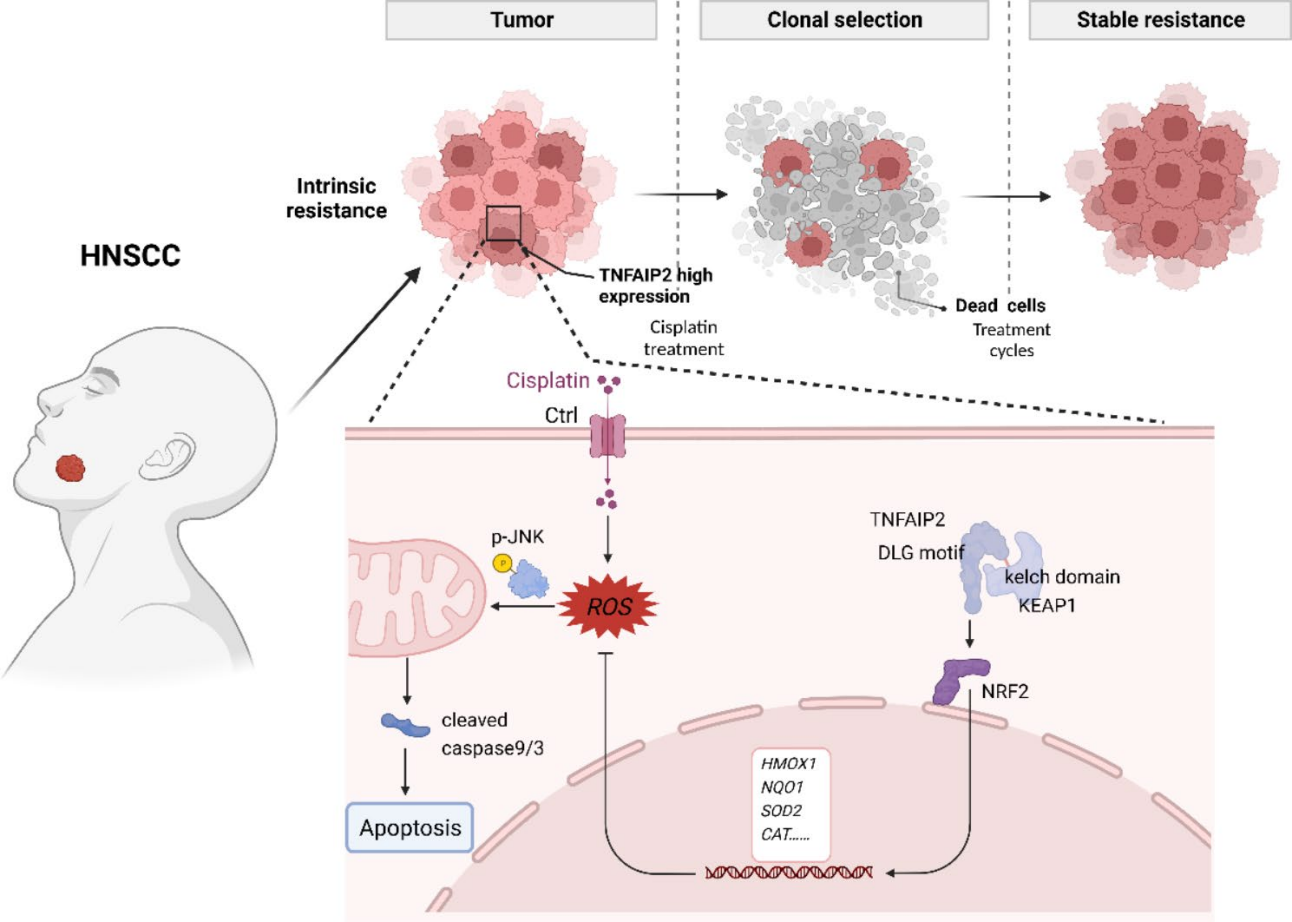
Schematic diagram of TNFAIP2 confers cisplatin resistance by sustaining NRF2 signaling activation in HNSCC. In cancer cells, highly expressed TNFAIP2 interacts with KEAP1, which protects NRF2 from ubiquitin proteasome-mediated degradation. Accumulated NRF2 occurs the nuclear translocation and promotes the transcription of antioxidant genes. These genes could efficiently scavenge ROS, which accumulate upon cisplatin treatment, thereby inhibiting the phosphorylation of JNK as well as the activation of its downstream apoptosis pathway. The above mechanism ultimately results in cisplatin treatment resistance in HNSCC patients with upregulation of TNFAIP2.

Collectively, the present study reveals the cisplatin treatment resistance-promoting role of TNFAIP2 in HNSCC. Our results demonstrate that TNFAIP2 can stabilize NRF2 by competitively interacting with KEAP1, thereby inhibiting cisplatin-induced oxidative stress and apoptosis, which ultimately confers cisplatin resistance (Fig. 8). TNFAIP2 might be a potential treatment target in improving the chemotherapy treatment effect in HNSCC, especially for the cisplatin-resistant subgroup.

## Electronic supplementary material

Below is the link to the electronic supplementary material.

**Additional file 1. Figure S1.** (a) TNFAIP2 mRNA expression between HNSCC and normal tissues from the TCGA. (b) Kaplan‒Meier survival curve of HNSCC patients with low versus high TNFAIP2 expression from the TCGA. (c) Kaplan‒Meier survival curve of HNSCC patients with low versus high TNFAIP2 expression from the GEO (GSE65858). (d) TNFAIP2 protein expression in HNSCC patients with or without TPF chemotherapy. (e) Correlations between TNFAIP2 mRNA expression and Taxol and 5-fluorouracil IC50 in 10 HNSCC cell lines. (f) Western blot analysis of the efficiency of TNFAIP2 overexpression/knockdown in HNSCC cell lines. (g) CCK-8 assays of cell viability in TNFAIP2-overexpressing HNSCC cell lines with or without cisplatin treatment. (h-i) Colony formation and qualification in TNFAIP2-overexpressing FADU (h) and CAL33 (i) cells with or without cisplatin treatment. (j-k) Cisplatin IC50 evaluation in TNFAIP2-knockdown FADU (j) and CAL33 (k). (l) Tumor weight of each group after the indicated treatments. Data are presented as the mean ± SEM. n.s., not significant; *** *P* < 0.001. **Figure S2**. (a) Flow cytometry analyses of cell cycle percentage in TNFAIP2-overexpressing HNSCC cell lines. (b) RT‒qPCR analysis of cisplatin resistance-associated genes in TNFAIP2-overexpressing HNSCC cell lines. (c-d) Flow cytometry analyses of ROS in TNFAIP2-knockdown FADU (c) and CAL33 cells (d). (e) Analyses of GSH/GSSG and SOD in TNFAIP2-knockdown HNSCC cell lines. (f-g) Flow cytometry analyses of cisplatin-induced apoptosis in TNFAIP2-overexpressing FADU (f) and CAL33 (g) cells. (h-i) Cisplatin IC50 evaluations in TNFAIP2-knockdown FADU (h) and CAL33 (i) cells with or without the JNK pathway inhibitor SP600125 (20 µmol/L, 2 h). Data are presented as the mean ± SEM. n.s., not significant; *** *P* < 0.001. **Figure S3.** (a) Relative luciferase activity in TNFAIP2-knockdown HNSCC cell lines transfected with ARE dual-luciferase reporter plasmids. (b) Relative luciferase activity in TNFAIP2-overexpressing HNSCC cell lines transfected with ARE dual-luciferase reporter plasmids. (c-d) The mRNA (c) and protein (d) levels of NRF2 target genes in TNFAIP2-knockdown HNSCC cell lines. (e-f) The mRNA (e) and protein (f) levels of NRF2 in TNFAIP2-knockdown HNSCC cell lines. Data are presented as the mean ± SEM. n.s., not significant; *** *P* < 0.001. AREs, antioxidant response elements. **Figure S4.** (a) Twelve unique peptides of KEAP1 identified by LC‒MS. (b) Relative luciferase activity in TNFAIP2-overexpressing HNSCC cell lines transfected with ARE dual-luciferase reporter plasmids with or without KEAP1 knockdown. (c-d) The mRNA (c) and protein (d) levels of KEAP1 and p62 in TNFAIP2-knockdown HNSCC cell lines. (e) Co-IP assay of the TNFAIP2 and NRF2 interaction in HNSCC cell lines. Data are presented as the mean ± SEM. n.s., not significant; *** *P* < 0.001. **Figure S5.** (a) Relative luciferase activity in TNFAIP2-knockdown HNSCC cell lines transfected with ARE luciferase reporter plasmids and rescued by wild-type TNFAIP2 or the N328-520 fragment. (b) RT‒qPCR analysis of NRF2 target genes in TNFAIP2-knockdown HNSCC cell lines rescued by wild-type TNFAIP2 or the N328-520 fragment. (c) Flow cytometry analyses of ROS in TNFAIP2-knockdown HNSCC cell lines rescued by transfection of TNFAIP2 with DLG deletion. (d) Relative luciferase activity in TNFAIP2-knockdown HNSCC cell lines transfected with ARE luciferase reporter plasmids and rescued by transfection of TNFAIP2 with DLG deletion. (e) RT‒qPCR analysis of NRF2 target genes in TNFAIP2-knockdown HNSCC cell lines rescued by transfection of TNFAIP2 with DLG deletion. (f-g) Cisplatin IC50 evaluations in TNFAIP2-knockdown FADU (f) and CAL33 cells (g) rescued by transfection of TNFAIP2 with DLG deletion. Data are presented as the mean ± SEM. n.s., not significant; *** *P* < 0.001. **Table S1.** Demographic and clinical characteristics of HNSCC patients. **Table S2.** Univariate and multivariate Cox analyses of HNSCC patients. **Table S3.** Target sequences of siRNAs in this study. **Table S4.** Plasmids used in this study. **Table S5.** Primary and secondary antibodies used in this study. **Table S6.** Primer for amplifying the human transcripts used in this study. **Table S7.** Critical commercial reagents used in this study.

**Additional file 2**: The results of coimmunoprecipitation coupled with mass spectrometry.

**Additional file 3**: The specific conditions for silver staining and LC‒MS as well as database searching parameters.

**Additional file 4**: Full unedited images of Western blots.

## Data Availability

The datasets supporting the conclusions of this article are included within the article and its additional files. Publicly available datasets analyzed in this study can be found here: https://portal.gdc.cancer.gov/, https://www.ncbi.nlm.nih.gov/gds/ (GSE65858, GSE39366).

## Abbreviations

HNSCC: head and neck squamous cell carcinoma
TNFAIP2: tumor necrosis factor alpha-induced protein 2
4NQO: 4-nitroquinoline N-oxide
ROS: reactive oxygen species
NFE2L2/NRF2: nuclear factor, erythroid 2-like 2
KEAP1: Kelch-like ECH-associated protein 1
TPF: Taxol + Platinum + Fluorouracil
MAPK1: mitogen-activated protein kinase 1
JNK: c-JUN N-terminal kinase
HIF1A: hypoxia-inducible factor 1-alpha
HE: hematoxylin-eosin
IHC: immunohistochemistry
ATCC: American Type Culture Collection
RT‒qPCR: real-time quantitative polymerase chain reaction
NAC: N-acetylcysteine
TCGA: The Cancer Genome Atlas
GEO: Gene Expression Omnibus
HO1: heme oxygenase 1
NQO1: NADPH quinone oxidoreductase 1
GCLC: glutamate-cysteine ligase catalytic subunit
CAT: catalase
SOD2: superoxide dismutase 2
CHX: cycloheximide
Co-IP: coimmunoprecipitation
Co-IP/MS: coimmunoprecipitation coupled with mass spectrometry
SQSTM1: sequestosome 1
AREs: antioxidant response elements
AUC: area under the curve
