# Supplementary material for: TNFAIP2 confers cisplatin resistance in head and neck squamous cell carcinoma via KEAP1/NRF2 signaling
Source: J Exp Clin Cancer Res. 2023 Aug 1;42:190. doi: 10.1186/s13046-023-02775-1 (PMC10391982; doi:10.1186/s13046-023-02775-1)
Supplement: Supplementary file 2 — Additional file 2: The results of coimmunoprecipitation coupled with mass spectrometry. [file 13046_2023_2775_MOESM2_ESM.docx]

**TNFAIP2 confers cisplatin resistance in head and neck squamous cell carcinoma via KEAP1/NRF2 signaling**

Teng Xu^1,2,3†^, Yuemei Yang^1,2,3†^, Zhihong Chen^1,2,3†^, Jinsong Wang^4^, Xiaolei Wang^4^, Yang Zheng^5^, Chao Wang^1,2,3^, Yachen Wang^1,2,3^, Zaiou Zhu^1^, Xu Ding^1^, Junbo Zhou^6^, Gang Li^7^, Hongchuang Zhang^8^, Wei Zhang^2,3*^, Yunong Wu^1,2,3*^, Xiaomeng Song^1,2,3*^

**Supplemental data**

5 Figures

7 Tables


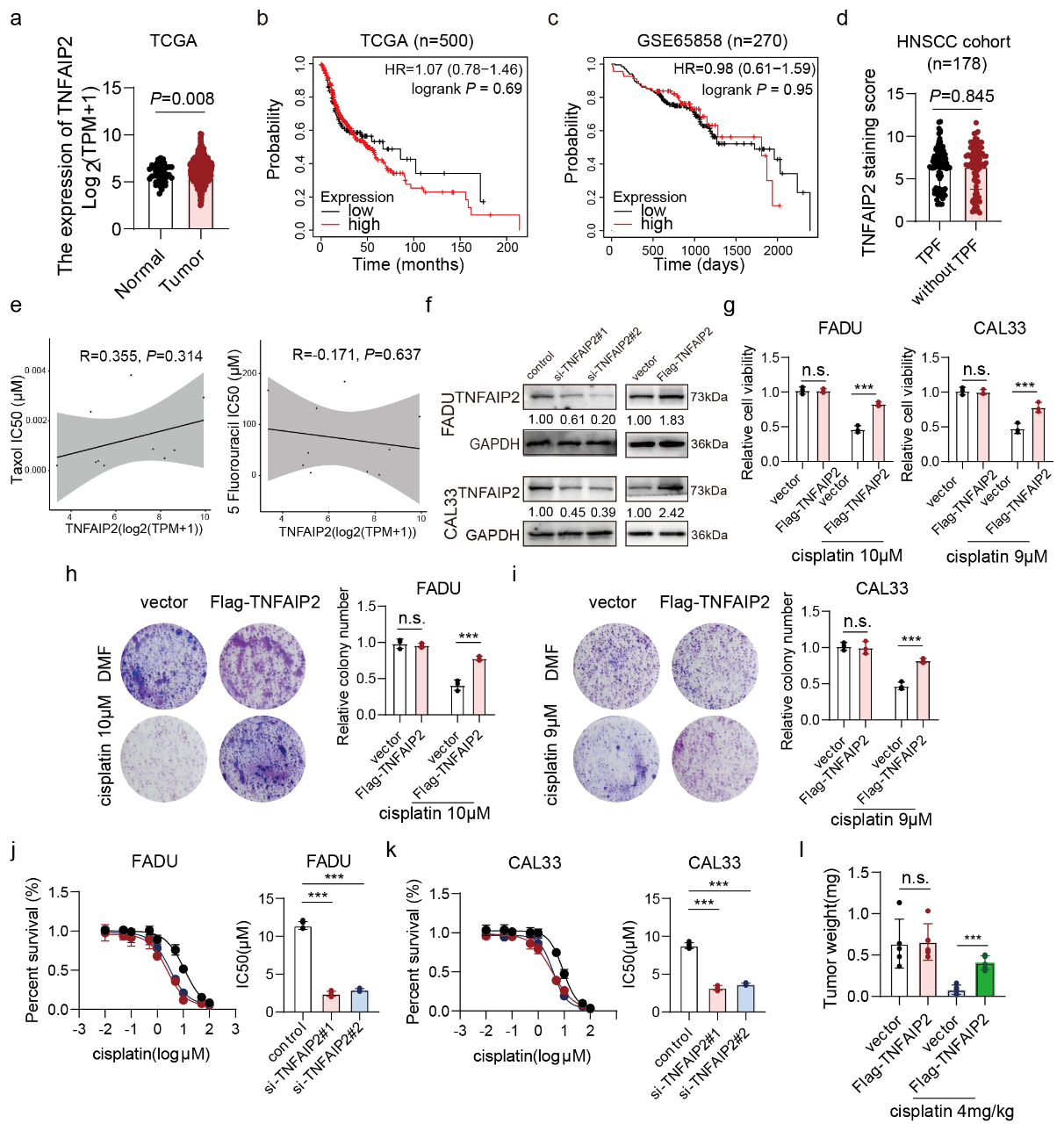


**Figure S1**. ***Patients with high TNFAIP2 expression tend to experience cisplatin treatment failure in HNSCC*** (a) TNFAIP2 mRNA expression between HNSCC and normal tissues from the TCGA. (b) Kaplan‒Meier survival curve of HNSCC patients with low versus high TNFAIP2 expression from the TCGA. (c) Kaplan‒Meier survival curve of HNSCC patients with low versus high TNFAIP2 expression from the GEO (GSE65858). (d) TNFAIP2 protein expression in HNSCC patients with or without TPF chemotherapy. (e) Correlations between TNFAIP2 mRNA expression and Taxol and 5-fluorouracil IC50 in 10 HNSCC cell lines. (f) Western blot analysis of the efficiency of TNFAIP2 overexpression/knockdown in HNSCC cell lines. (g) CCK-8 assays of cell viability in TNFAIP2-overexpressing HNSCC cell lines with or without cisplatin treatment. (h-i) Colony formation and qualification in TNFAIP2-overexpressing FADU (h) and CAL33 (i) cells with or without cisplatin treatment. (j-k) Cisplatin IC50 evaluation in TNFAIP2-knockdown FADU (j) and CAL33 (k). (l) Tumor weight of each group after the indicated treatments. Data are presented as the mean ± SEM. n.s., not significant; *** *P*<0.001.


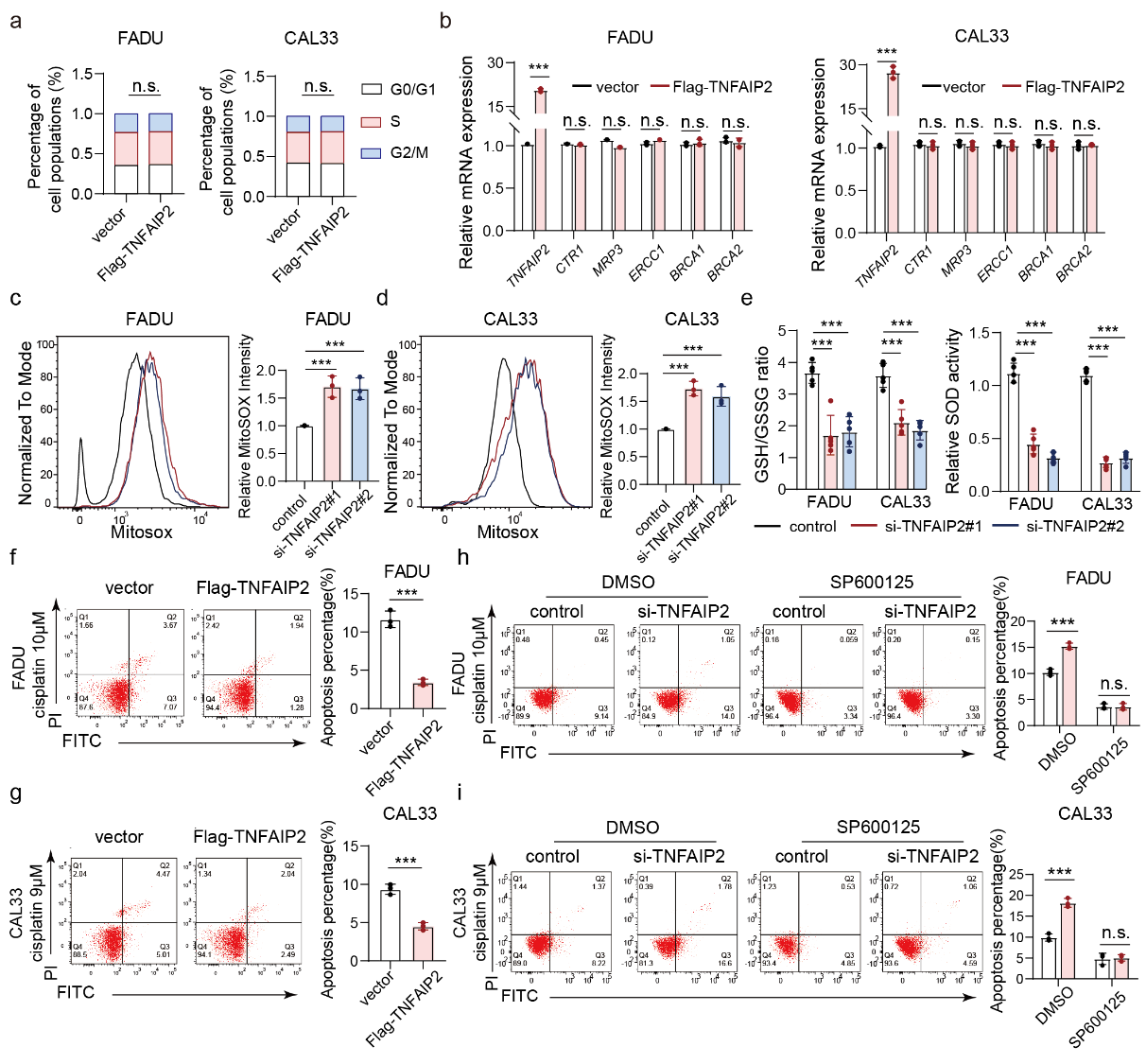


**Figure S2.** ***TNFAIP2 protects HNSCC cells from cisplatin-induced apoptosis by inhibiting ROS/JNK signaling*** (a) Flow cytometry analyses of cell cycle percentage in TNFAIP2-overexpressing HNSCC cell lines. (b) RT‒qPCR analysis of cisplatin resistance-associated genes in TNFAIP2-overexpressing HNSCC cell lines. (c-d) Flow cytometry analyses of ROS in TNFAIP2-knockdown FADU (c) and CAL33 cells (d). (e) Analyses of GSH/GSSG and SOD in TNFAIP2-knockdown HNSCC cell lines. (f-g) Flow cytometry analyses of cisplatin-induced apoptosis in TNFAIP2-overexpressing FADU (f) and CAL33 (g) cells. (h-i) Cisplatin IC50 evaluations in TNFAIP2-knockdown FADU (h) and CAL33 (i) cells with or without the JNK pathway inhibitor SP600125 (20 μmol/L, 2 h). Data are presented as the mean ± SEM. n.s., not significant; *** *P*<0.001.


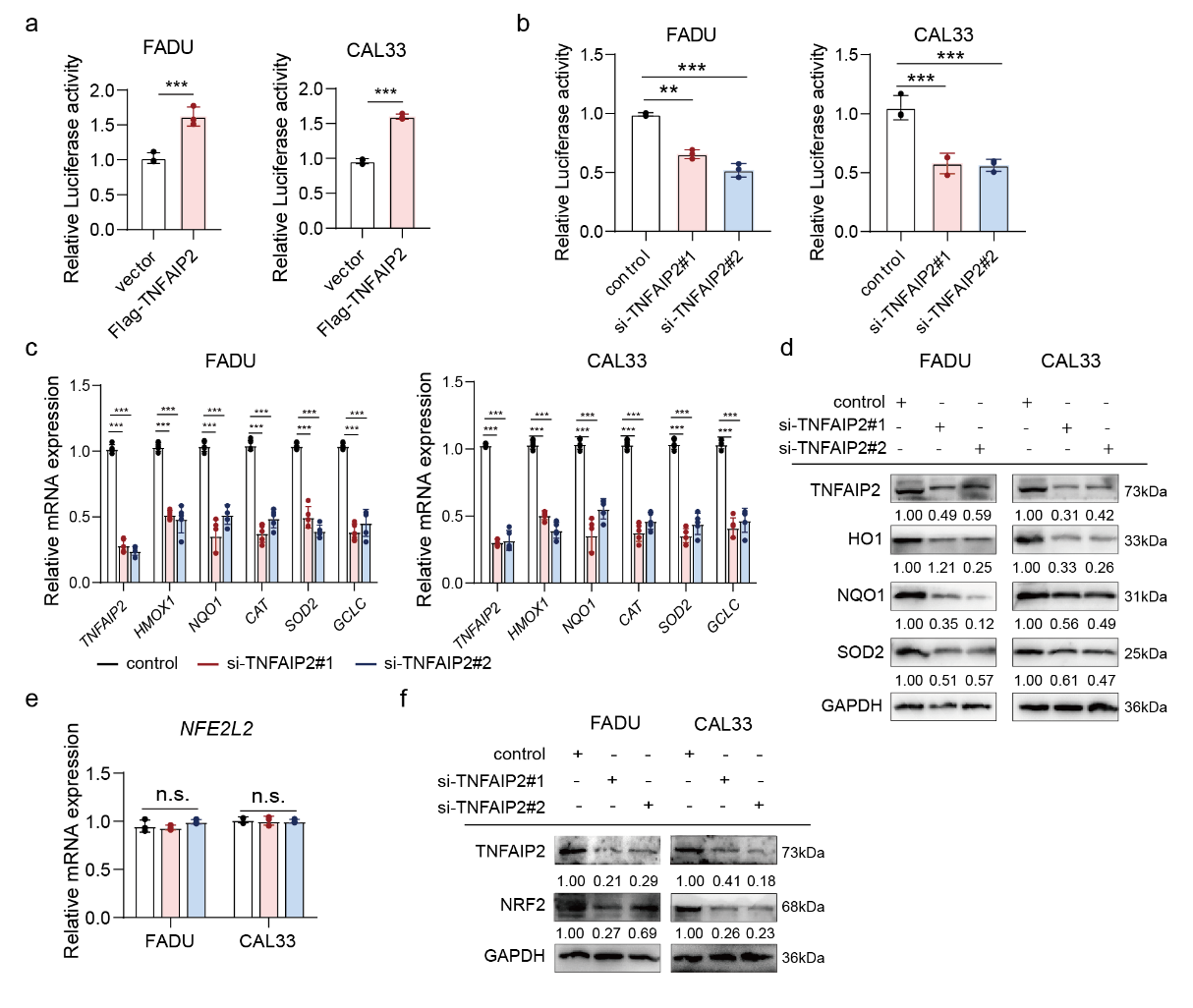


**Figure S3. *TNFAIP2 stabilizes the NRF2 protein by inhibiting its ubiquitination and degradation*** (a) Relative luciferase activity in TNFAIP2-knockdown HNSCC cell lines transfected with ARE dual-luciferase reporter plasmids. (b) Relative luciferase activity in TNFAIP2-overexpressing HNSCC cell lines transfected with ARE dual-luciferase reporter plasmids. (c-d) The mRNA (c) and protein (d) levels of NRF2 target genes in TNFAIP2-knockdown HNSCC cell lines. (e-f) The mRNA (e) and protein (f) levels of NRF2 in TNFAIP2-knockdown HNSCC cell lines. Data are presented as the mean ± SEM. n.s., not significant; *** *P*<0.001. AREs, antioxidant response elements.


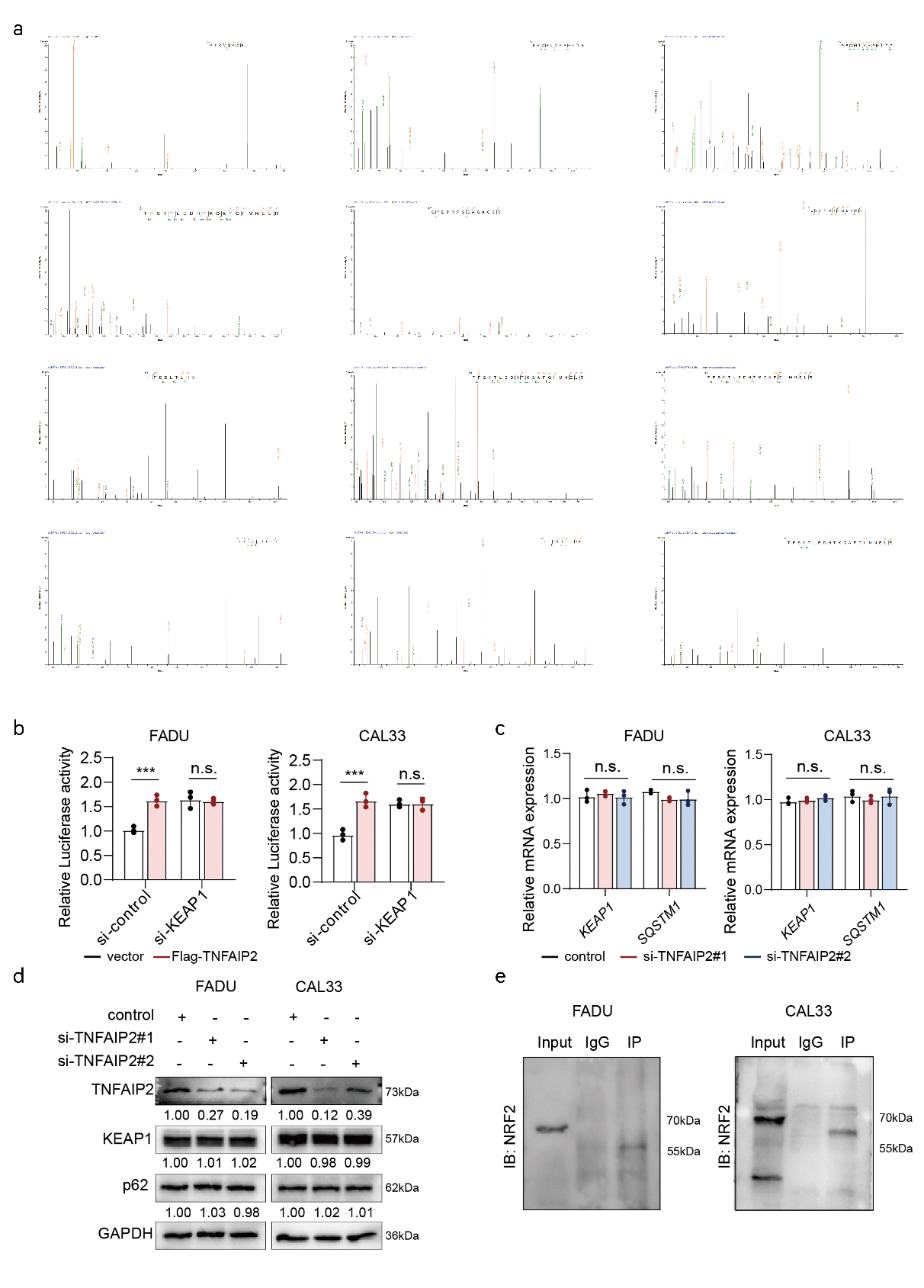


**Figure S4. *TNFAIP2 interacts with KEAP1 to stabilize NRF2*** (a) Twelve unique peptides of KEAP1 identified by LC‒MS. (b) Relative luciferase activity in TNFAIP2-overexpressing HNSCC cell lines transfected with ARE dual-luciferase reporter plasmids with or without KEAP1 knockdown. (c-d) The mRNA (c) and protein (d) levels of KEAP1 and p62 in TNFAIP2-knockdown HNSCC cell lines. (e) Co-IP assay of the TNFAIP2 and NRF2 interaction in HNSCC cell lines. Data are presented as the mean ± SEM. n.s., not significant; *** *P*<0.001.


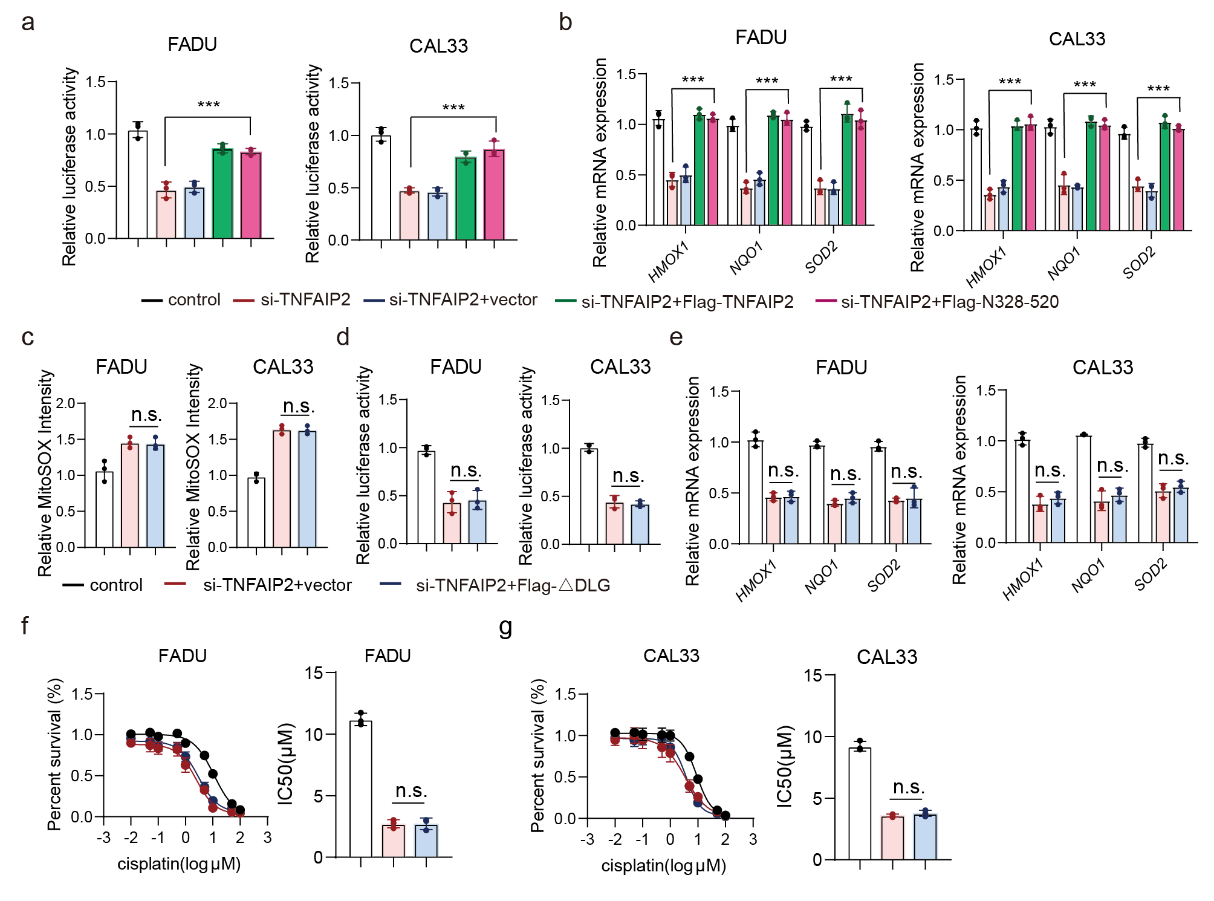


**Figure S5.** ***The DLG motif and Kelch domain mediate TNFAIP2 interaction with KEAP1*** (a) Relative luciferase activity in TNFAIP2-knockdown HNSCC cell lines transfected with ARE luciferase reporter plasmids and rescued by wild-type TNFAIP2 or the N328-520 fragment. (b) RT‒qPCR analysis of NRF2 target genes in TNFAIP2-knockdown HNSCC cell lines rescued by wild-type TNFAIP2 or the N328-520 fragment. (c) Flow cytometry analyses of ROS in TNFAIP2-knockdown HNSCC cell lines rescued by transfection of TNFAIP2 with DLG deletion. (d) Relative luciferase activity in TNFAIP2-knockdown HNSCC cell lines transfected with ARE luciferase reporter plasmids and rescued by transfection of TNFAIP2 with DLG deletion. (e) RT‒qPCR analysis of NRF2 target genes in TNFAIP2-knockdown HNSCC cell lines rescued by transfection of TNFAIP2 with DLG deletion. (f-g) Cisplatin IC50 evaluations in TNFAIP2-knockdown FADU (f) and CAL33 cells (g) rescued by transfection of TNFAIP2 with DLG deletion. Data are presented as the mean ± SEM. n.s., not significant; *** *P*<0.001.

**Table 1.** Demographic and clinical characteristics of HNSCC patients (n=178)

|  | Whole cohort | | TNFAIP2 high expression | | TNFAIP2 low expression | | *P* value |
| --- | --- | --- | --- | --- | --- | --- | --- |
|  | (n=178) | | (n=108) | | (n=70) | |  |
| Sex |  |  |  |  |  |  | 0.084 |
| Male | 125 | 70.2% | 81 | 75.0% | 44 | 62.9% |  |
| Female | 53 | 29.8% | 27 | 25.0% | 26 | 37.1% |  |
| Age |  |  |  |  |  |  | 0.017* |
| <65 y | 101 | 56.7% | 69 | 63.9% | 32 | 45.7% |  |
| >=65 y | 77 | 43.3% | 39 | 36.1% | 38 | 54.3% |  |
| Primary site |  |  |  |  |  |  | 0.070 |
| Oral cavity | 129 | 72.5% | 73 | 67.6% | 56 | 80.0% |  |
| Pharynx | 49 | 27.5% | 35 | 32.4% | 14 | 20.0% |  |
| Pathological grade |  |  |  |  |  |  | 0.659 |
| Well | 42 | 23.6% | 28 | 25.9% | 14 | 20.0% |  |
| Moderate | 82 | 46.1% | 48 | 44.4% | 34 | 48.6% |  |
| Poor | 54 | 30.3% | 32 | 29.6% | 22 | 31.4% |  |
| T stage |  |  |  |  |  |  | 0.001* |
| T1 | 56 | 31.5% | 28 | 25.9% | 28 | 40.0% |  |
| T2 | 64 | 36.0% | 33 | 30.6% | 31 | 44.3% |  |
| T3-T4 | 58 | 32.6% | 47 | 43.5% | 11 | 15.7% |  |
| N stage |  |  |  |  |  |  | <0.001* |
| N0 | 82 | 46.1% | 38 | 35.2% | 44 | 62.9% |  |
| N1-N3 | 96 | 53.9% | 70 | 64.8% | 26 | 37.1% |  |
| AJCC stage |  |  |  |  |  |  | <0.001* |
| I | 24 | 13.5% | 8 | 7.4% | 16 | 22.9% |  |
| II | 43 | 24.2% | 18 | 16.7% | 25 | 35.7% |  |
| III-IV | 111 | 62.4% | 82 | 75.9% | 29 | 41.4% |  |
| TPF Chemotherapy |  |  |  |  |  |  | 0.576 |
| No | 86 | 48.3% | 54 | 50.0% | 32 | 45.7% |  |
| Yes | 92 | 51.7% | 54 | 50.0% | 38 | 54.3% |  |

Data are presented as n (%). *, statistical significance (*P*<0.05). HNSCC, head and neck squamous cell carcinoma; AJCC, American Joint Committee on Cancer; TPF, Taxol + platinum + fluorouracil

**Table 2.** Univariate and multivariate Cox analyses of HNSCC patients (n=178)

|  | Univariate analysis | |  | Multivariate analysis | |  |
| --- | --- | --- | --- | --- | --- | --- |
|  | HR | 95% CI | *P* value | HR | 95% CI | *P* value |
| Sex |  |  |  |  |  |  |
| Male | 1.000 |  |  |  |  |  |
| Female | 1.245 | 0.813-1.906 | 0.314 |  |  |  |
| Age |  |  |  |  |  |  |
| <65 y | 1.000 |  |  |  |  |  |
| >=65 y | 0.842 | 0.563-1.261 | 0.405 |  |  |  |
| Primary Site |  |  |  |  |  |  |
| Oral cavity | 1.000 |  |  |  |  |  |
| Pharynx | 1.309 | 0.855-2.006 | 0.215 |  |  |  |
| Pathological grade |  |  |  |  |  |  |
| Well | 1.000 |  |  | 1.000 |  |  |
| Moderate | 1.291 | 0.741-2.249 | 0.367 | 1.511 | 0.860-2.654 | 0.151 |
| Poor | 2.119 | 1.211-3.707 | 0.009* | 2.207 | 1.236-3.941 | 0.007* |
| T stage |  |  |  |  |  |  |
| T1 | 1.000 |  |  | 1.000 |  |  |
| T2 | 1.911 | 1.081-3.376 | 0.026* | 1.549 | 0.758-3.164 | 0.230 |
| T3-T4 | 3.061 | 1.769-5.297 | <0.001* | 1.485 | 0.816-2.705 | 0.196 |
| N stage |  |  |  |  |  |  |
| N0 | 1.000 |  |  | 1.000 |  |  |
| N1-N3 | 2.211 | 1.445-3.382 | <0.001* | 1.272 | 0.632-2.560 | 0.500 |
| AJCC stage |  |  |  |  |  |  |
| I | 1.000 |  |  | 1.000 |  |  |
| II | 6.644 | 1.553-28.431 | 0.011* | 7.28 | 1.692-31.333 | 0.008* |
| III-IV | 11.949 | 2.931-48.715 | 0.001* | 9.929 | 2.412-40.863 | 0.001* |
| TPF chemotherapy |  |  |  |  |  |  |
| No | 1.000 |  |  | 1.000 |  |  |
| Yes | 0.617 | 0.414-0.919 | 0.018* | 0.672 | 0.419-0.937 | 0.023* |
| TNFAIP2 level |  |  |  |  |  |  |
| Low | 1.000 |  |  | 1.000 |  |  |
| High | 2.294 | 1.446-3.639 | <0.001* | 1.844 | 1.139-2.983 | 0.013* |

*, statistical significance (*P*<0.05). HNSCC, head and neck squamous cell carcinoma; HR, hazard ratio; AJCC, American Joint Committee on Cancer; TPF, Taxol + platinum + fluorouracil

**Table S3.** Target sequences of siRNAs in this study

| **Name** | **Source** | **Sequences (5′ to 3′)** |
| --- | --- | --- |
| NC | GenePharma | UUCUCCGAACGUGUCACGUTT |
| TNFAIP2#1 | GenePharma | CCCAAUGACAUCAUCAACATT |
| TNFAIP2#2 | GenePharma | CCAGUGCCAUCAAGAUUGATT |
| Tnfaip2#1 | Ribobio | GCAAACTCCTGAGGAATTA |
| Tnfaip2#2(2’OMe/5’Col) | Ribobio | GCACCTGCACCTAGTGAAA |
| Tnfaip2#3 | Ribobio | GGAACATACTGGACATCAA |
| NRF2 | Hanbio Biotechnology | CUUGCAUUAAUUCGGGAUAUAdTdT |
| KEAP1 | Hanbio Biotechnology | GCGAAUGAUCACAGCAAUGAAdTdT |

**Table S4.** Plasmids used in this study

| **Plasmids** | **Sourse** |
| --- | --- |
| pCMV-MCS-GFP | Hanbio Biotechnology |
| pCMV-MCS-GFP-TNFAIP2-Flag | Hanbio Biotechnology |
| pCMV-MCS-GFP-(1-327)-TNFAIP2-Flag | Hanbio Biotechnology |
| pCMV-MCS-GFP-(328-654)-TNFAIP2-Flag | Hanbio Biotechnology |
| pCMV-MCS-GFP-(328--520)-TNFAIP2-Flag | Hanbio Biotechnology |
| pCMV-MCS-GFP-(521-654)-TNFAIP2-Flag | Hanbio Biotechnology |
| pCMV-MCS-RFP-DLG^381^ mutant TNFAIP2-Flag | Hanbio Biotechnology |
| pCMV-MCS-RFP-DLG^382^ mutant TNFAIP2-Flag | Hanbio Biotechnology |
| pCMV-MCS-RFP-∆DLG mutant TNFAIP2-Flag | Hanbio Biotechnology |
| pCMV-MCS-GFP-Keap1-His | Hanbio Biotechnology |
| pCMV-MCS-GFP-NB-Keap1-His | Hanbio Biotechnology |
| pCMV-MCS-GFP-IVR-Keap1-His | Hanbio Biotechnology |
| pCMV-MCS-GFP-KC-Keap1-His | Hanbio Biotechnology |
| pCMV-MCS-GFP-∆K-Keap1-His | Hanbio Biotechnology |
| pCMV-MCS-GFP-∆C-Keap1-His | Hanbio Biotechnology |
| pGL3-basic luciferase reporter plasmid | Ribobio |

**Table S5.** Primary and secondary antibodies used in this study

| **Antibodies** | **Source** | **Catalogue Number** |
| --- | --- | --- |
| Anti-TNFAIP2 | Santa Cruz | sc-28318 |
| Anti-NRF2 | Proteintech | 16396-1-AP |
| Anti-KEAP1 | Proteintech | 10503-2-AP |
| Anti-HO1 | Proteintech | 66743-1-Ig |
| Anti-NQO1 | Proteintech | 67240-1-Ig |
| Anti-Ki67 | Cell Signaling technology | 9449 |
| Anti-Ubiqutin | Abcam | ab179434 |
| Anti-p62 | Abmart | M047405 |
| Anti-JNK | Proteintech | 66210-1-Ig |
| Anti-p-JNK | Proteintech | 80024-1-RR |
| Anti-caspase9 | Cell Signaling technology | 9508 |
| Anti-cl-caspase9 | Cell Signaling technology | 9505 |
| Anti-caspase3 | Cell Signaling technology | 9662 |
| Anti-cl-caspase3 | Cell Signaling technology | 9664 |
| Anti-GAPDH | Proteintech | 10494-1-AP |
| Goat Anti-Mouse IgG H&L (Alexa Fluor 647) | Proteintech | SA00009-1 |
| Goat Anti-Rabbit IgG H&L (Alexa Fluor 647) | Proteintech | SA00003-2 |
| Anti-mouse IgG HRP-linked Ab | Proteintech | SA00001-1 |
| Anti-rabbit IgG HRP-linked Ab | Proteintech | SA00001-2 |
| MaxVision^TM^ HRP-Polymer anti-Mouse/Rabbit IHC Kit | MXB Biotechnologies | Kit-5010 |
| Mouse IgG | Cell Signaling technology | 7076P2 |
| Rabbit IgG | Cell Signaling technology | 7074P2 |

**Table S6.** Primer for amplifying the human transcripts used to in this study

| **Gene** | **Source** | **Sequences (5′ to 3′)** |
| --- | --- | --- |
| TNFAIP2 | General Biology | F: GTACGAGCTGCTGAGGGATC  R: AGCAGAGATCCCAGTCCCAT |
| CTR1 | General Biology | F: TTACCCGCATACCAAGGAGAA  R: TGGGCAGAACTGATAGGACAATA |
| MRP3 | General Biology | F: TGGGGTGAAGTTTCGTACTGG  R: CACGTTTGACTGAGTTGGTGATA |
| ERCC1 | General Biology | F: CCTTATTCCGATCTACACAGAGC  R: TATTCGGCGTAGGTCTGAGGG |
| BRCA1 | General Biology | F: GAAACCGTGCCAAAAGACTTC  R: CCAAGGTTAGAGAGTTGGACAC |
| BRCA2 | General Biology | F: CACCCACCCTTAGTTCTACTGT  R: CCAATGTGGTCTTTGCAGCTAT |
| HMOX1 | General Biology | F: AAGACTGCGTTCCTGCTCAAC  R: AAAGCCCTACAGCAACTGTCG |
| NQO1 | General Biology | F: GAAGAGCACTGATCGTACTGGC  R: GAAGAGCACTGATCGTACTGGC |
| CAT | General Biology | F: TGGAGCTGGTAACCCAGTAGG  R: CCTTTGCCTTGGAGTATTTGGTA |
| SOD2 | General Biology | F: GCTCCGGTTTTGGGGTATCTG  R: GCGTTGATGTGAGGTTCCAG |
| GCLC | General Biology | F: GGAGGAAACCAAGCGCCAT  R: CTTGACGGCGTGGTAGATGT |
| NFE2L2/NRF2 | General Biology | F: TCAGCGACGGAAAGAGTATGA  R: CCACTGGTTTCTGACTGGATGT |
| KEAP1 | General Biology | F: CTGGAGGATCATACCAAGCAGG  R: GGATACCCTCAATGGACACCAC |
| SQSTM1/p62 | General Biology | F: GCACCCCAATGTGATCTGC  R: CGCTACACAAGTCGTAGTCTGG |
| GAPDH | General Biology | F: GGAGCGAGATCCCTCCAAAAT  R: GGCTGTTGTCATACTTCTCATGG |
| *Tnfaip2-mouse* | General Biology | F: AGGAGGAGTCTGCGAAGAAGA  R: GGCAGTGGACCATCTAACTCG |
| *Gapdh-mouse* | General Biology | F: AGGTCGGTGTGAACGGATTTG  R: GGGGTCGTTGATGGCAACA |

**Table S7.** Critical commercial reagents used to in this study

| **Chemicals** | **Source** | **Catalogue Number** |
| --- | --- | --- |
| ExFect Transfection Reagent | Vazyme | T101-AA |
| MitoSOX^TM^ Green mitochondrial superoxide indicator | Thermo Fisher Scientific | M36006 |
| DMF (N, N-Dimethylformamide) | MedChemExpress | HY-Y0345 |
| CHX (Cycloheximide) | MedChemExpress | HY-12320 |
| MG-132 | Beyotime | S1748 |
| MLN4924 | MedChemExpress | HY-70062 |
| Protein A/G β PLUS Agarose beads | Santa Cruz | sc-2003 |
| NAC(N-Acetylcysteine) | Sigma | A7250 |
| SP600125 | MedChemExpress | HY-12041 |
| Cisplatin | APExBIO | A821 |
| 4NQO | Sigma | N8141-5G |
| DAPI (40,6-Diamidino-2-phenylindole dihydrochloride) | Sigma | D9542 |
| Trizol | Vazyme | R401-01 |
| 5×HiScript II qRT SuperMix | Vazyme | R222-01-AB |
| 2× ChamQ Universal SYBR qPCR Master Mix | Vazyme | Q711-02-AA |
| Dual Luciferase Reporter Assay kit | Beyotime | RG088S |
| TUNEL kit | KeyGEN biotechnology | KGA702-B |
| Cell counting kit-8 | APExBIO | K1018 |
| GSH/GSSG Ratio Detection Assay Kit II | Abcam | ab205811 |
| SOD Detection Assay Kit | Jiancheng | A001-3-2 |
| Goat serum | BOSTER | AR0009 |
