# Supplementary material for: TNFAIP2 confers cisplatin resistance in head and neck squamous cell carcinoma via KEAP1/NRF2 signaling
Source: J Exp Clin Cancer Res. 2023 Aug 1;42:190. doi: 10.1186/s13046-023-02775-1 (PMC10391982; doi:10.1186/s13046-023-02775-1)
Supplement: Supplementary file 3 — Additional file 3: The specific conditions for silver staining and LC‒MS as well as database searching parameters. [file 13046_2023_2775_MOESM3_ESM.docx]

**TNFAIP2 confers cisplatin resistance in head and neck squamous cell carcinoma via KEAP1/NRF2 signaling**

Teng Xu^1,2,3†^, Yuemei Yang^1,2,3†^, Zhihong Chen^1,2,3†^, Jinsong Wang^4^, Xiaolei Wang^4^, Yang Zheng^5^, Chao Wang^1,2,3^, Yachen Wang^1,2,3^, Zaiou Zhu^1^, Xu Ding^1^, Junbo Zhou^6^, Gang Li^7^, Hongchuang Zhang^8^, Wei Zhang^2,3*^, Yunong Wu^1,2,3*^, Xiaomeng Song^1,2,3*^

**The specific conditions for silver staining and LC-MS as well as database searching parameters**

**In-solution degestion**

The samples were mixed with 200μl of UA buffer (8M Urea, 150mM TrisHCl pH8.0), loaded into the filter devices and centrifuged at 14,000g for 15min. The concentrates were diluted in the device with 200μl of UA buffer and centrifuged again. Then the concentrates were mixed with 100μl of 50mM IAA in UA buffer and incubated in darkness at room temperature for 30min followed by centrifugation for 15min. Then, the concentrate was diluted with100ul of UA buffer, and concentrated again. This step was repeated twice. The concentrate was diluted with 100μl of 25mM NH4HCO3 and concentrated again. This step was repeated twice. The resulting concentrate was diluted to 40 µL of 25mM NH4HCO3 containing 2 µg of Trypsin. After overnight incubation at 37℃, peptides were collected by centrifugation of the ﬁlter units for 20 min.

**LC-MS/MS**

Experiments were performed on a Q Exactive mass spectrometer that was coupled to Easy nLC (Thermo Fisher Scientific). The peptide mixture was loaded onto a the C18-reversed phase column (15 cm long, 75 μm inner diameter) packed in-house with RP-C18 5μm resin in buffer A (0.1% Formic acid in HPLC-grade water) and separated with a linear gradient of buffer B (0.1% Formic acid in 84% acetonitrile ) at a flow rate of 250 nl/min controlled by IntelliFlow technology over 60 min. MS data was acquired using a data-dependent top10 method dynamically choosing the most abundant precursor ions from the survey scan (300–1800 m/z) for HCD fragmentation. Determination of the target value is based on predictive Automatic Gain Control (pAGC). Dynamic exclusion duration was 20 s. Survey scans were acquired at a resolution of 70,000 at m/z 200 and resolution for HCD spectra was set to 17,500 at m/z 200. Normalized collision energy was 27 eV and the underfill ratio, which specifies the minimum percentage of the target value likely to be reached at maximum fill time, was defined as 0.1%. The instrument was run with peptide recognition mode enabled.

**Sequence Database Searching and Data Analysis**

The MS data were analyzed using MaxQuant software version 1.3.0.5. MS data were searched against the uniprot_homo_202249_20211008 (202249 total entries, downloaded 2021/10/8). An initial search was set at a precursor mass window of 6 ppm. The search followed an enzymatic cleavage rule of Trypsin/P and allowed maximal two missed cleavage sites and a mass tolerance of 20ppm for fragment ions. Carbamidomethylation of cysteines was defined as fixed modification, while protein N-terminal acetylation and methionine oxidation were defined as variable modifications for database searching. The cutoff of global false discovery rate (FDR) for peptide and protein identification was set to 0.01.
