# Supplementary material for: TNFAIP2 confers cisplatin resistance in head and neck squamous cell carcinoma via KEAP1/NRF2 signaling
Source: J Exp Clin Cancer Res. 2023 Aug 1;42:190. doi: 10.1186/s13046-023-02775-1 (PMC10391982; doi:10.1186/s13046-023-02775-1)

Full unedited gel for Figure 2K

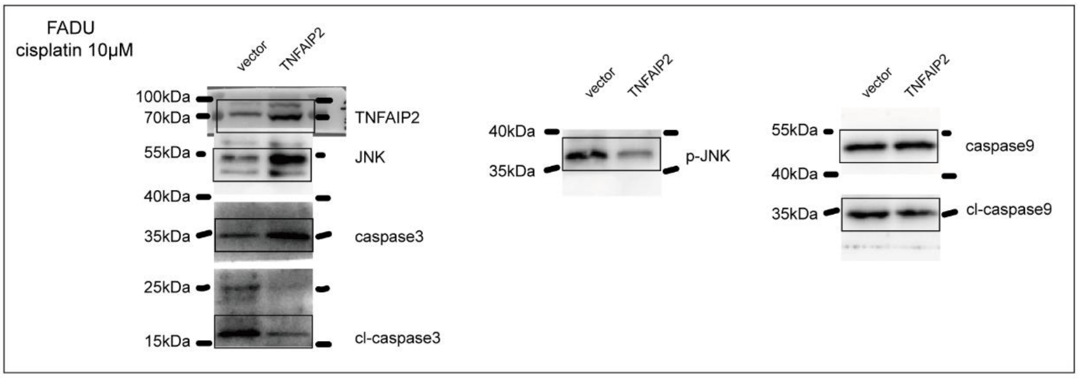

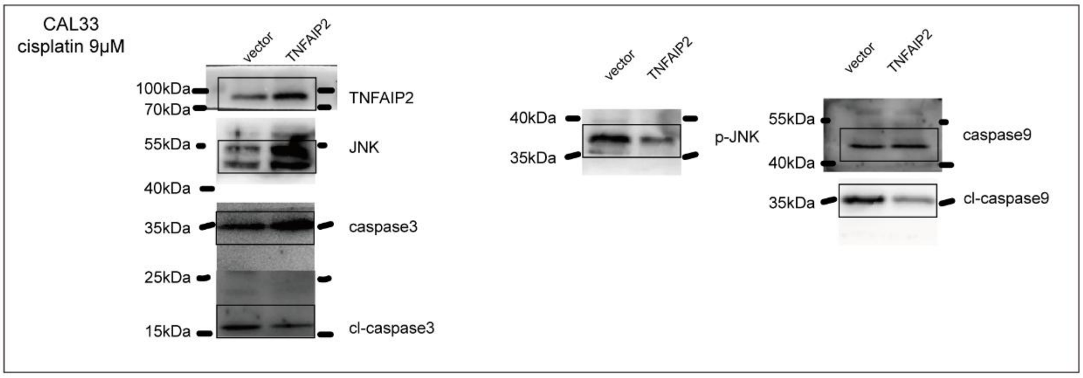

Full unedited gel for Figure 2L

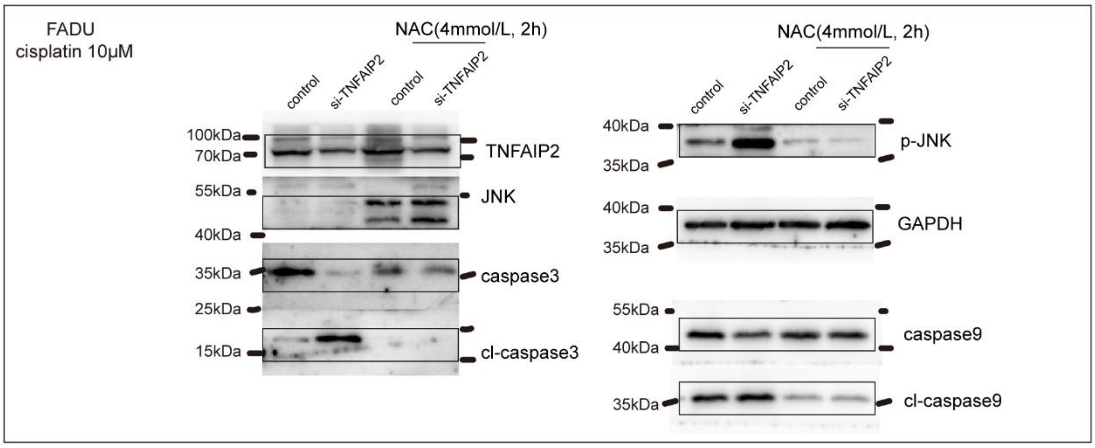

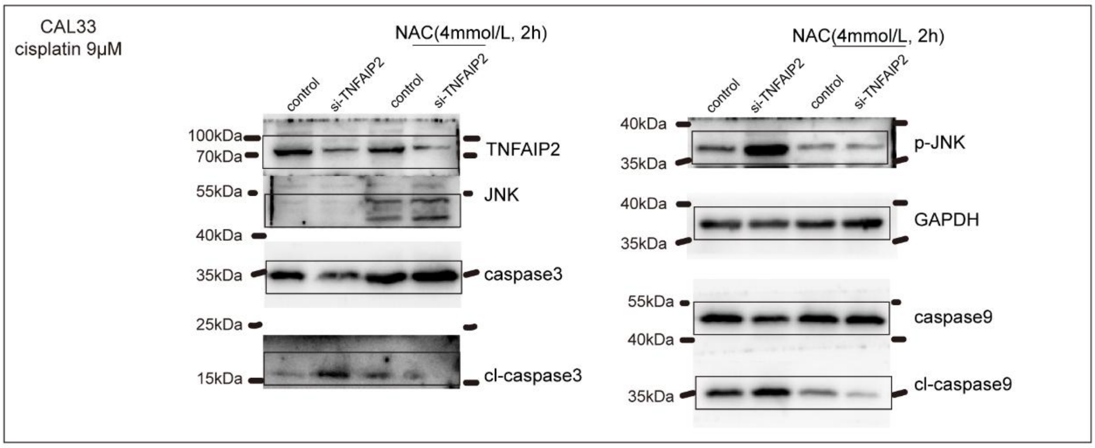

Full unedited gel for Figure 3G

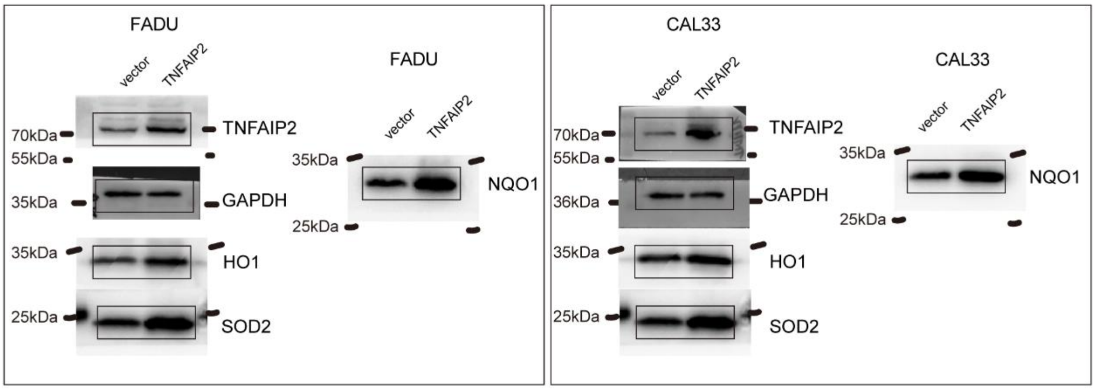

Full unedited gel for Figure 3I

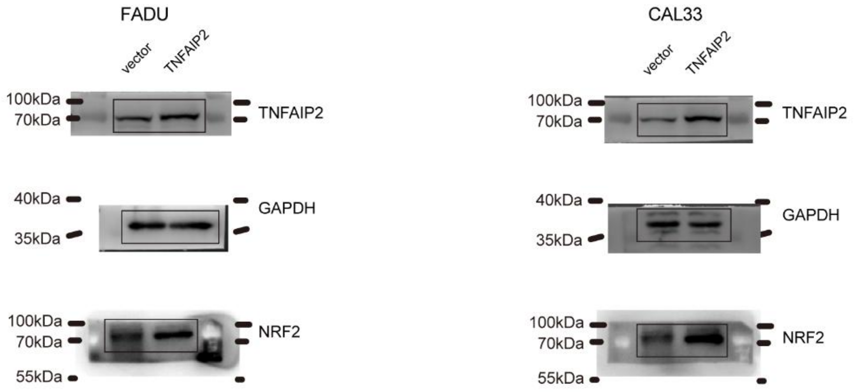

Full unedited gel for Figure 3J

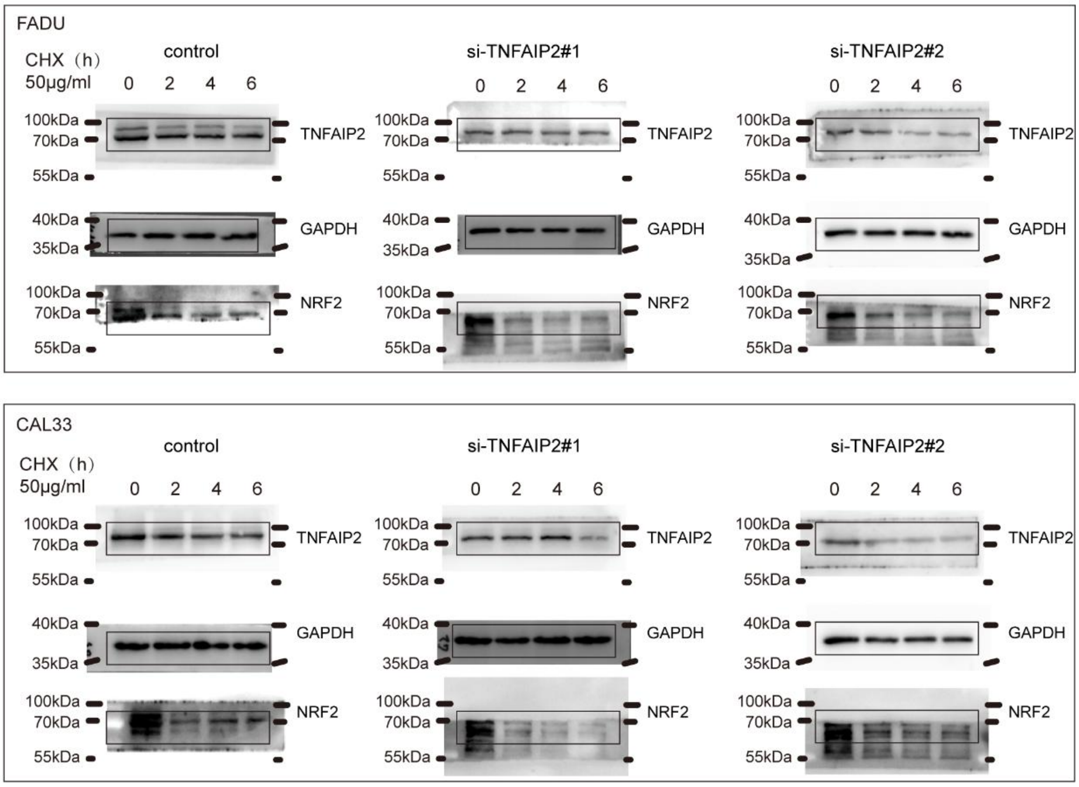

Full unedited gel for Figure 3K

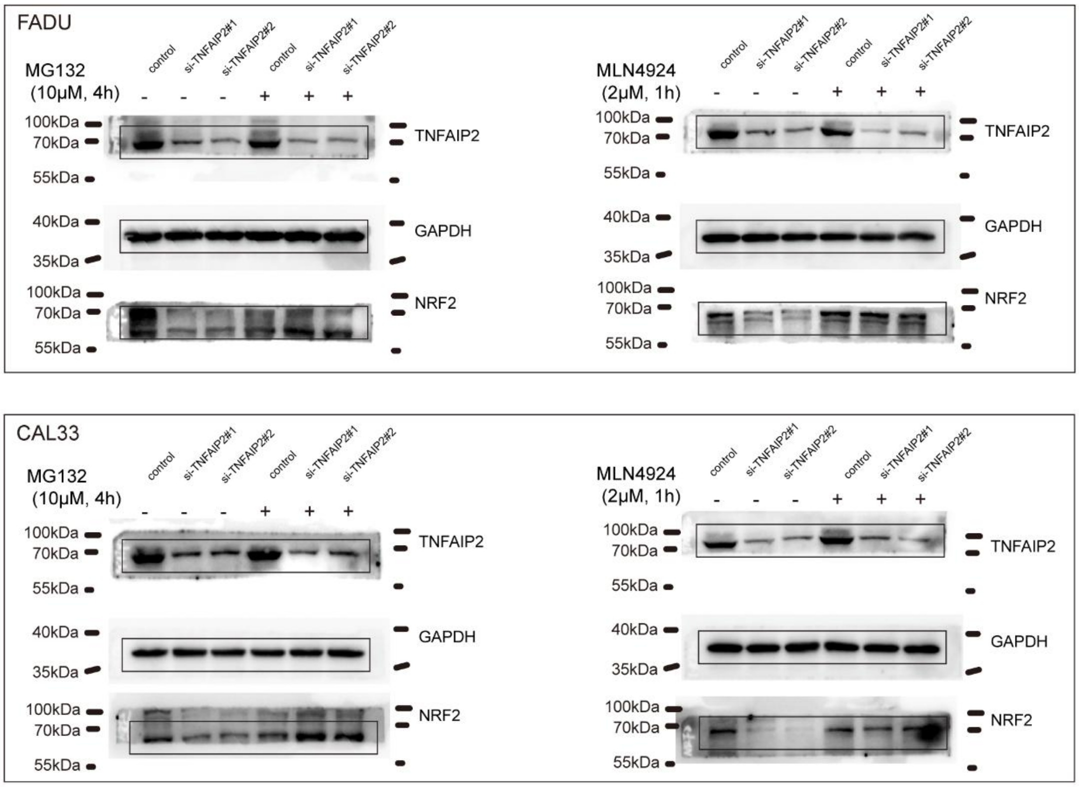

Full unedited gel for Figure 3L

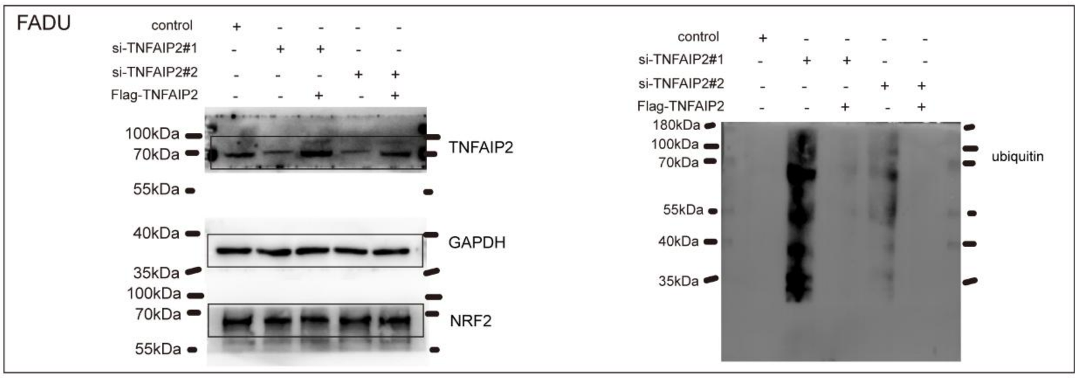

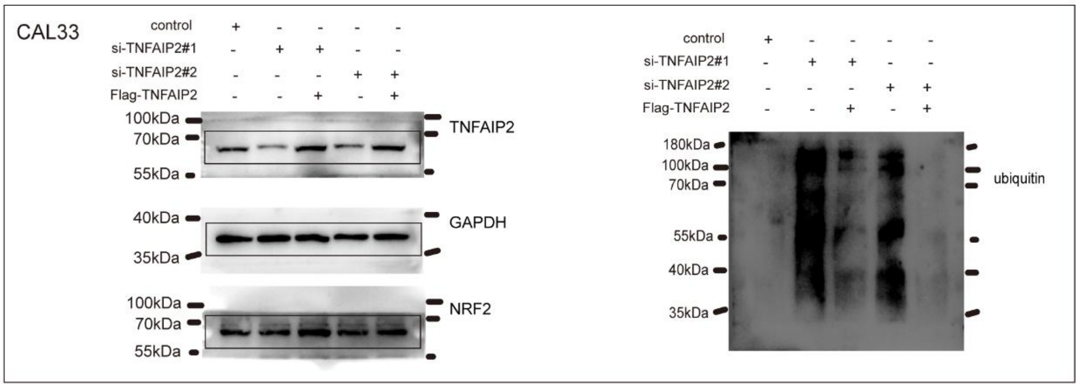

Full unedited gel for Figure 4B

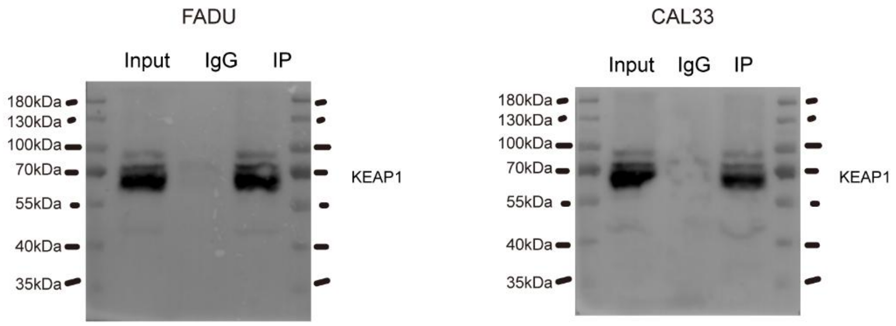

Full unedited gel for Figure 4F

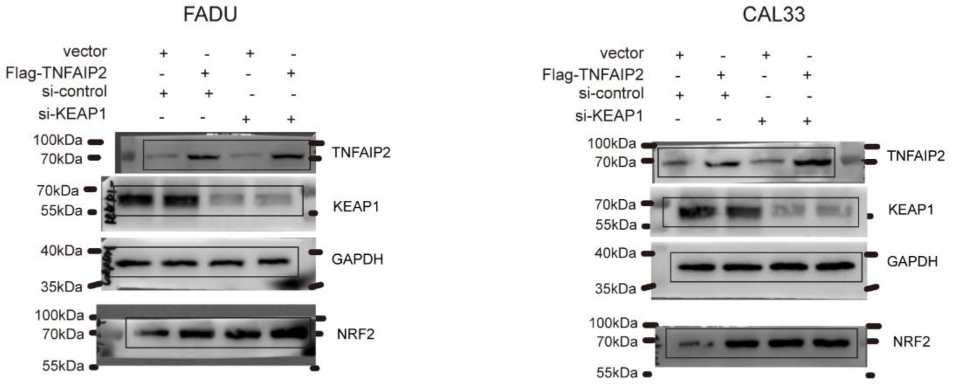

Full unedited gel for Figure 4G

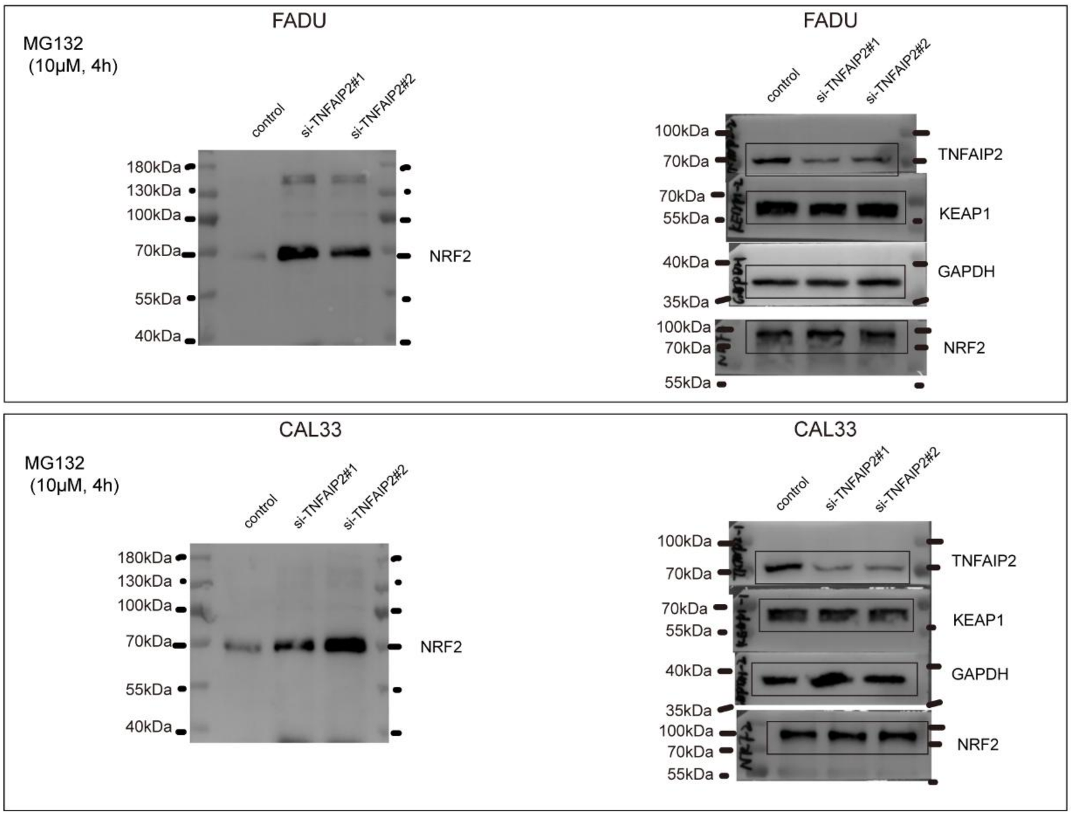

Full unedited gel for Figure 4H

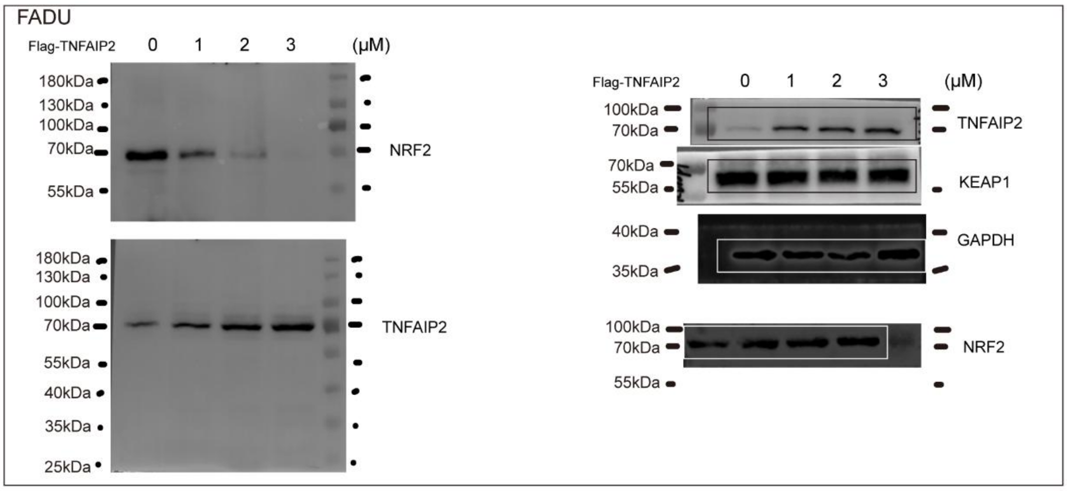

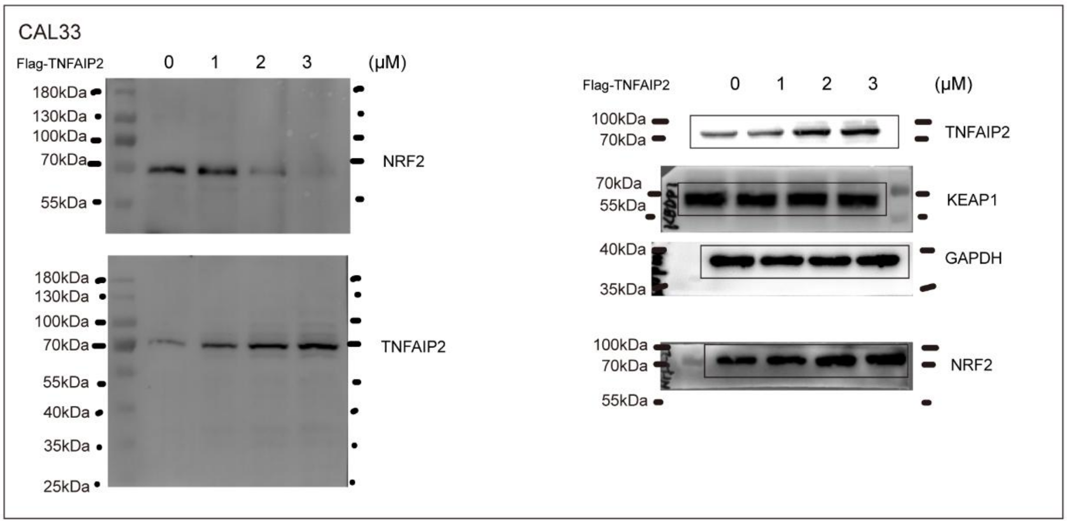

Full unedited gel for Figure 5B

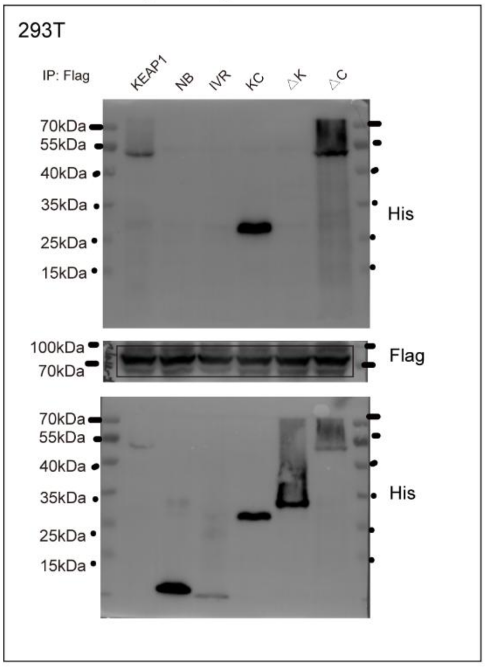

Full unedited gel for Figure 5C

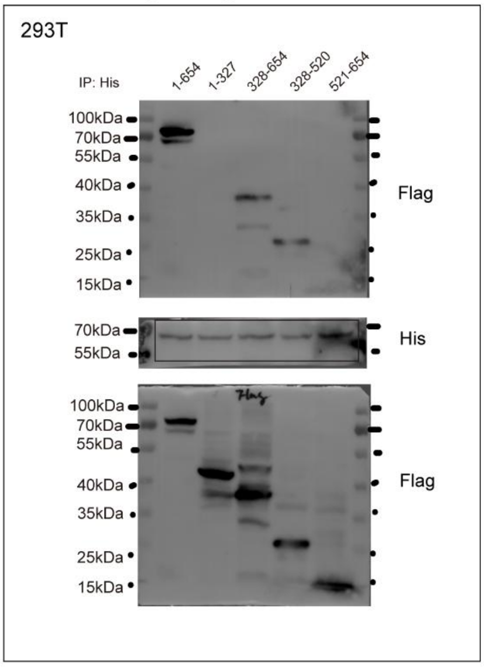

Full unedited gel for Figure 5E

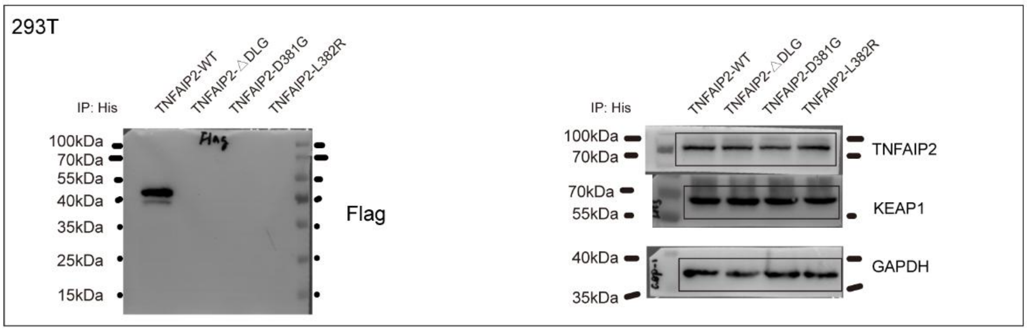

Full unedited gel for Figure 5F

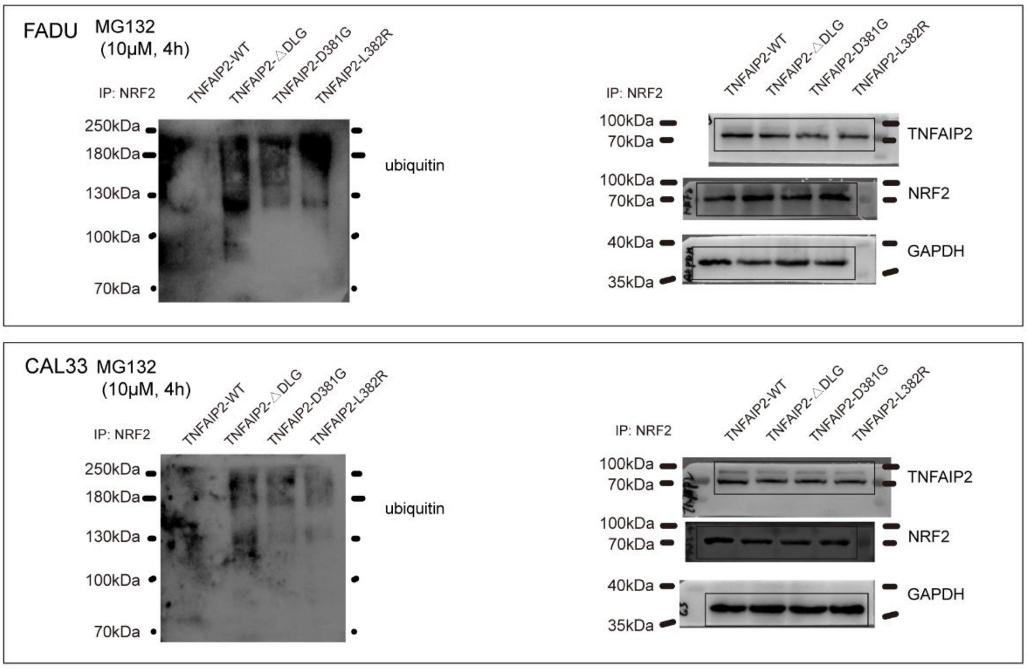

Full unedited gel for Figure 5J

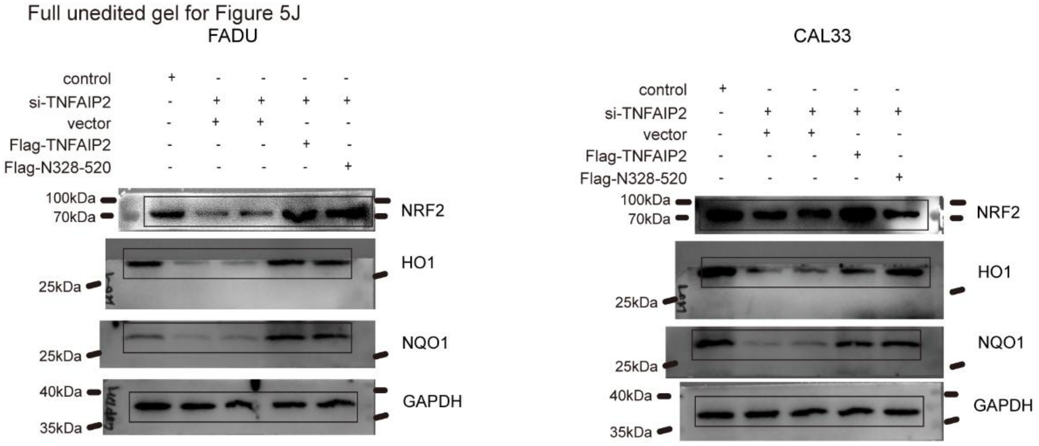

Full unedited gel for Figure 7A   N, Nmoral tissue   T, Tumor tissue

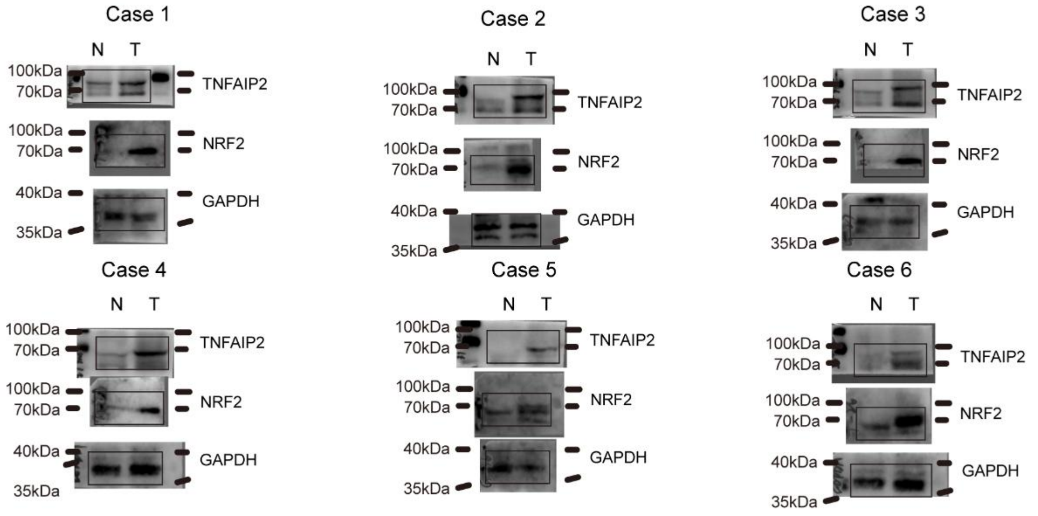

Full unedited gel for Figure S1B

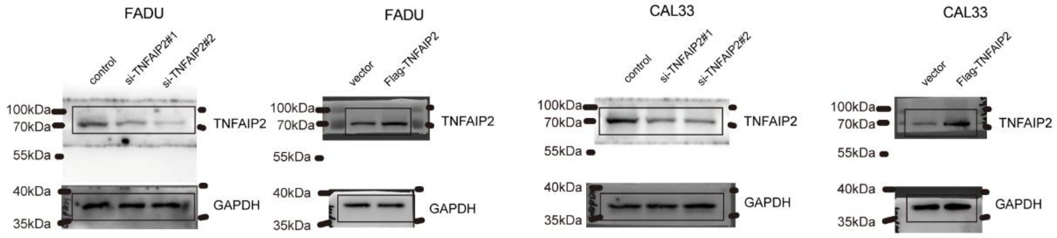

Full unedited gel for Figure S3D

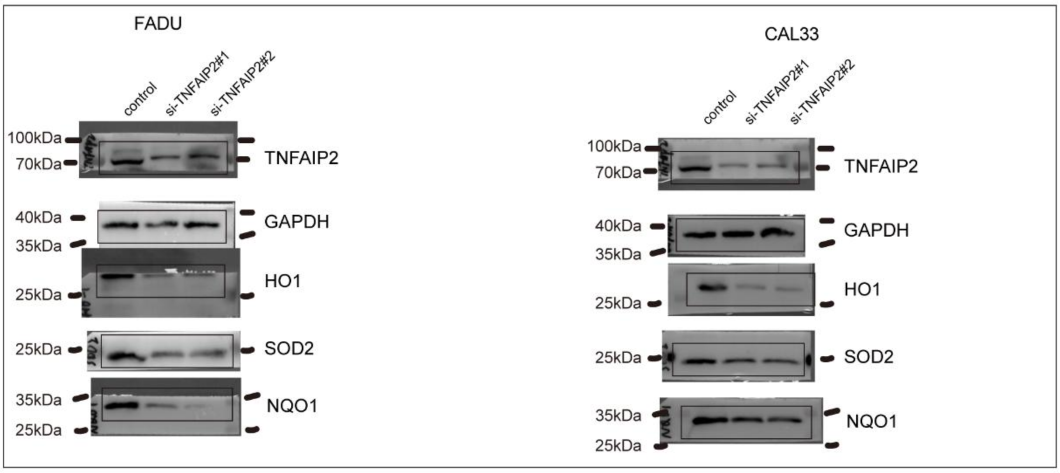

Full unedited gel for Figure S3F

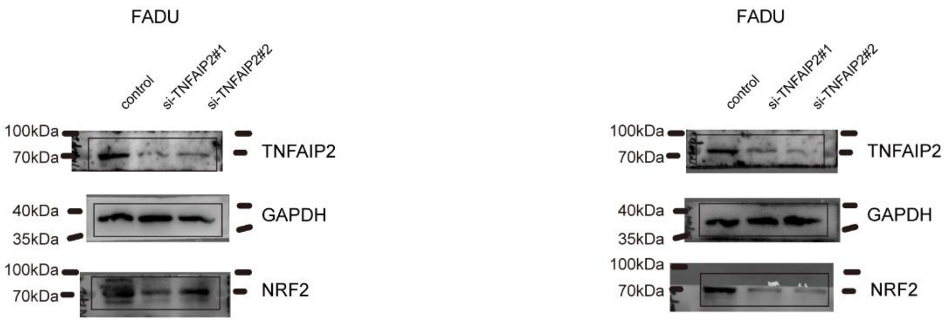

Full unedited gel for Figure S4C

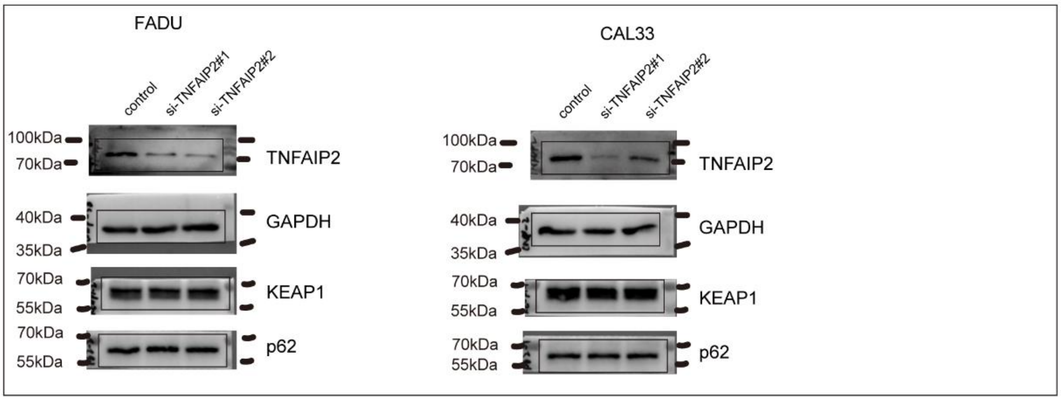

Full unedited gel for Figure S4D

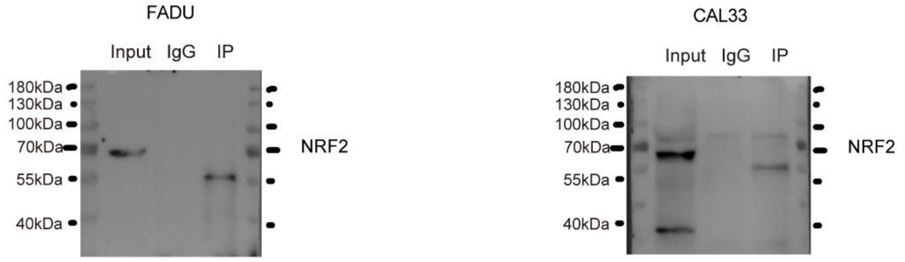

Supplement: Supplementary file 4 — Additional file 4: Full unedited images of Western blots. [file 13046_2023_2775_MOESM4_ESM.pdf]
